# Tailoring Light–Matter Interactions in Overcoupled Resonator for Biomolecule Recognition and Detection

**DOI:** 10.1007/s40820-024-01520-3

**Published:** 2024-09-26

**Authors:** Dongxiao Li, Hong Zhou, Zhihao Ren, Cheng Xu, Chengkuo Lee

**Affiliations:** 1 Department of Electrical and Computer Engineering, National University of Singapore, Singapore, 117583 Singapore; 2 Center for Intelligent Sensors and MEMS (CISM), National University of Singapore, Singapore, 117608 Singapore

**Keywords:** Plasmonic nanoantennas, Light-matter interaction, Surface-enhanced infrared absorption, Overcoupled, Biosensing

## Abstract

Plasmonic nanoantennas provide unique opportunities for precise control of light–matter coupling in surface-enhanced infrared absorption (SEIRA) spectroscopy, but most of the resonant systems realized so far suffer from the obstacles of low sensitivity, narrow bandwidth, and asymmetric Fano resonance perturbations. Here, we demonstrated an overcoupled resonator with a high plasmon-molecule coupling coefficient (μ) (OC-Hμ resonator) by precisely controlling the radiation loss channel, the resonator-oscillator coupling channel, and the frequency detuning channel. We observed a strong dependence of the sensing performance on the coupling state, and demonstrated that OC-Hμ resonator has excellent sensing properties of ultra-sensitive (7.25% nm^−1^), ultra-broadband (3–10 μm), and immune asymmetric Fano lineshapes. These characteristics represent a breakthrough in SEIRA technology and lay the foundation for specific recognition of biomolecules, trace detection, and protein secondary structure analysis using a single array (array size is 100 × 100 µm^2^). In addition, with the assistance of machine learning, mixture classification, concentration prediction and spectral reconstruction were achieved with the highest accuracy of 100%. Finally, we demonstrated the potential of OC-Hμ resonator for SARS-CoV-2 detection. These findings will promote the wider application of SEIRA technology, while providing new ideas for other enhanced spectroscopy technologies, quantum photonics and studying light–matter interactions.

**Supplementary Information:**

The online version contains supplementary material available at 10.1007/s40820-024-01520-3.

## Highlights

- Proposed a new paradigm for nanoantenna design using coupled-mode theory.
- Designed an OC-Hµ resonator with excellent sensing performance.
- Using OC-Hµ resonators for biomolecule recognition and detection.

**Supplementary Information:**

The online version contains supplementary material available at 10.1007/s40820-024-01520-3.

## Graphical abstract

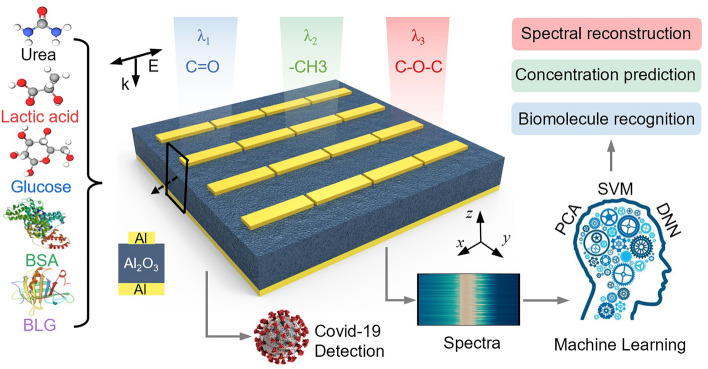

## Introduction

Maximizing tailored light–matter interactions in nanoscale materials is a central goal of nanophotonics [1]. Resonant nanosystems have been demonstrated for the confinement and control of electromagnetic energy in subwavelength volumes, providing unique opportunities for enhanced light–matter interactions [2]. Based on this property of nanophotonics, many applications have been demonstrated, including enhanced spectroscopy [3–5], nonlinear optics [6], plasmon catalysis [7], quantum optics [8] and nanolasers [9]. Among them, surface-enhanced infrared absorption (SEIRA) spectroscopy is more attractive [10, 11]. This is because the mid-infrared spectrum contains infrared vibrational fingerprints of various biochemical molecules, which are related to molecular composition, chemical bonds, and inherent configurations. The emergence of nanophotonics has solved the limitation of low sensitivity of infrared in detecting trace molecules or ultra-thin film systems [12–15]. SEIRA spectroscopy has made significant progress recently and has achieved molecular dynamic monitoring [16–19], hyperspectral imaging [20–24], and biochemical molecule detection [25–27], showing a wide range of application potential. Despite significant progress in controlling the spectral composition of light–matter interactions, several thorny issues have hindered widespread application of this technology.

Low sensitivity, narrow bandwidth, and asymmetric Fano resonance are the three main obstacles restricting the wide application of SEIRA [28–31]. Among them, low sensitivity leads to unsound value of the limit of detection (LOD). Narrow bandwidth affects the versatility of individual devices [31]. The asymmetric Fano resonance hinders the direct acquisition of molecular fingerprint vibration information [30]. Huge efforts have been made to solve the above problems. First, a series of strategies, such as utilizing nanogaps [28, 32, 33], molecular enrichment [27, 34], and bound states in the continuum (BIC) [20], have been proposed to improve the sensitivity of SEIRA. Second, methods such as multiresonance [17, 35, 36], supercell [31], array [20], modulation [25, 37] and gradient metasurfaces [38, 39] were used to collect broadband spectral data. In terms of weakening asymmetric Fano resonance, methods such as broadband [31], envelope [20, 40–42] or secondary calibration [43] have been proposed to restore the natural absorption fingerprint of molecules. While the above approaches all address one or two of the obstacles to some extent, simultaneously addressing all three of the above obstacles using a single array device remains a huge challenge. Solving these three challenges simultaneously on a single array device facilitates smaller device size, higher integration, and broader applicability.

Recently, there has been widespread interest in exploring nanoantennas driven by physics to achieve exceptional sensing capabilities. This pursuit has led to the emergence of various novel resonance modes with high-performance sensing characteristics, such as Fano-resonant [26], BIC [20], exceptional points [44], and ε-near-zero nanocavities [33]. Temporal coupled-mode theory (TCMT), serving as the foundational physics framework for describing light–matter interactions [22, 29, 45], has emerged as a promising approach to drive the design and optimization of nanoantennas. This method holds tremendous potential in advancing our understanding and utilization of nanoantennas, thereby facilitating the development of novel sensing platforms with enhanced performance. Currently, TCMT has been widely used in the exploration of resonator-molecule coupling sensitivity, including single-layer nanoantennas [31, 46, 47], metamaterial absorbers (MA) [48–50] and surface plasmon–phonon polaritons [51]. Importantly, an overcoupled (OC) resonator can be obtained by improving the ratio of radiative loss (γ_r_) to absorptive loss (γ_a_) of the MA [48]. Research has shown that OC resonators have wide bandwidth and the potential to eliminate asymmetric Fano resonance. Although increasing the radiation loss can achieve the desired bandwidth, increasing the radiation loss without restriction can also lead to sensitivity degradation problems. Therefore, it is crucial to carefully tailor the light–matter interaction in the OC resonator and balance various aspects of SEIRA performance in the process.

Here, we precisely controlled light–matter interactions in OC resonators based on TCMT, and obtained an ultra-sensitive, ultra-bandwidth, and immune asymmetric Fano resonance sensing platform on a single array. Under the theoretical framework constructed by TCMT, we revealed that the SEIRA sensitivity in the resonator can be precisely controlled through three channels. The three channels are the radiation loss channel, the resonator-oscillator coupling channel and the frequency detuning channel, respectively. The precise design of the radiation loss channel allows the resonator to be tuned from undercoupled (UC) to OC state. The resonator-oscillator coupling channel can manipulate the switching of the resonator-molecule coupling system between weak and strong coupling states. Frequency detuning channel is used to access maximum sensitivity at specific coupling states. Through precise control of multiple channels, we constructed a resonator with high radiation loss and high resonator-oscillator coupling coefficient, which we called OC-Hμ. The OC-Hμ resonator can be in an OC state and a strong coupling state at the same time, and the superposition of these two states exhibits excellent sensing performance. Using polymethyl methacrylate (PMMA) as the probe molecule, we demonstrated that the OC-Hμ resonator has the advantages of ultra-sensitive (7.25% nm^−1^), ultra-broadband (3–10 μm), and immune asymmetric Fano resonance. In particular, the sensitivity of OC-Hμ is improves by 4.1 times compared with traditional OC resonators. Based on the above three advantages, it further extends the three advantages of lower LOD, spectral versatility and spectral anti-distortion. These advantages represent a major advancement in SEIRA technology and will promote its widespread applications in the field of molecular detection. As a concept demonstration, we implemented the detection of five different biomolecules and the mixtures detection of two proteins based on a single array. With the assistance of machine learning (ML), we achieved classification of multiple biomolecules and concentration prediction of mixed proteins. After removing Fano interference, the secondary structure of the enhanced spectrum remains consistent with the secondary structure of the sample itself. This property paves the way for accurate resolution of the secondary structure of proteins. Finally, we demonstrated the potential of OC-Hμ resonator for SARS-CoV-2 detection.

## Experimental Section

### Sample Preparation

PMMA was used as a molecular probe for SEIRA performance demonstration. 0.2% 495 K PMMA in anisole was spin-coated onto the device surface with speed of 4000 rpm for 1 min. Then the device was baked on a hotplate at 180 °C for 3 min. Urea (Sigma-Aldrich, U5128), lactic acid (Sigma-Aldrich, 252,476), glucose (Sigma-Aldrich, G8270), bovine serum albumin (BSA, Sigma-Aldrich, A7030), β-lactoglobulin (BLG, Sigma-Aldrich, L3908) were used to demonstrate the spectral versatility of OC-Hµ. Prepared solutions of different concentrations in deionized water (urea: 60 ng µL^−1^, lactic acid: 6 ng µL^−1^, glucose: 60 ng µL^−1^, BSA: 50 ng µL^−1^, BLG: 50 ng µL^−1^). In protein quantitative measurements, BSA and BLG concentrations ranged from 10 to 250 ng µL^−1^. In protein secondary structure measurements, the protein concentration loaded onto OC-Hµ and UC-Hµ devices was 100 ng µL^−1^. A single array of OC-Hµ was used for spectral versatility, protein concentration measurement and secondary structure analysis. For each measurement, use a micropipette to extract 2 µL of the solution and drop it on the device. Measurements were taken after the droplets had dried for 15 min. After each measurement, the sample was immersed in deionized water for 5 min to remove the sample. As an additional cleaning step, the devices were cleaned in a plasma cleaner (Femto Science VITA 8) for 4 min to remove any residual material.

### Apparatus

Fourier transform infrared (FTIR) spectrometer (Cary 660, Agilent Technologies) was coupled with an infrared microscope of mercury cadmium telluride (Cary 610, Agilent Technologies) cooled by liquid nitrogen to collect the infrared spectra of the devices. The signal acquisition area was limited to a single 100 × 100 µm^2^ array by the knife-edge aperture. The measured spectrum of the Au mirror was used as the background spectrum. Other parameter settings include a resolution of 4 cm^−1^, and the average value was taken after 16 scans for each measurement. Scanning electron microscope (SEM, Hitachi Regulus 8230) was used to characterize the device’s morphology.

### Data Processing and Machine Learning

The preprocessing of spectral data, baseline calibration, parameter fitting, molecular vibration signal extraction, data post-processing and principal component analysis (PCA) were all completed by Origin (Origin-Lab Corporation, USA) software. Wavelength (ω_L_, unit: μm) and wavenumber (ω_N_, unit: cm^−1^) are converted by the formula: ω_N_ = 10,000/ω_L_. The proposed support vector machine (SVM) classifiers were developed on Python 3.6 using the scikit-learn package. Since SVM is a classification algorithm for a two-group classification problem, we transform it into a set of binary classification problems to distinguish different molecules or mixtures. Among them, 80% of the experimental data was the training set and 20% of the experimental data was the test set. The linear kernel function was used as the kernel of the SVM. We need to consider the value of the parameter C when training an SVM with a linear kernel. Here we set the C parameter to 1.0. In terms of concentration prediction, we also developed the MM-DNN model based on Python 3.6 using the scikit-learn software package. MM-DNN used Keras to define a sequential model to perform the regression task of two output nodes. The model architecture consists of an input layer, three hidden layers, and an output layer. The input layer was configured with 1687 nodes, corresponding to spectral data points. Each hidden layer had 64 neurons and used the ReLU activation function. The output layer had two neurons and used a linear activation function. The model was compiled using the Adam optimizer, a mean squared error (MSE) loss function, and metrics including mean absolute error and accuracy.

## Results and Discussion

### Working Mechanism of OC-Hµ Sensing Platform

**Fig. 1:**
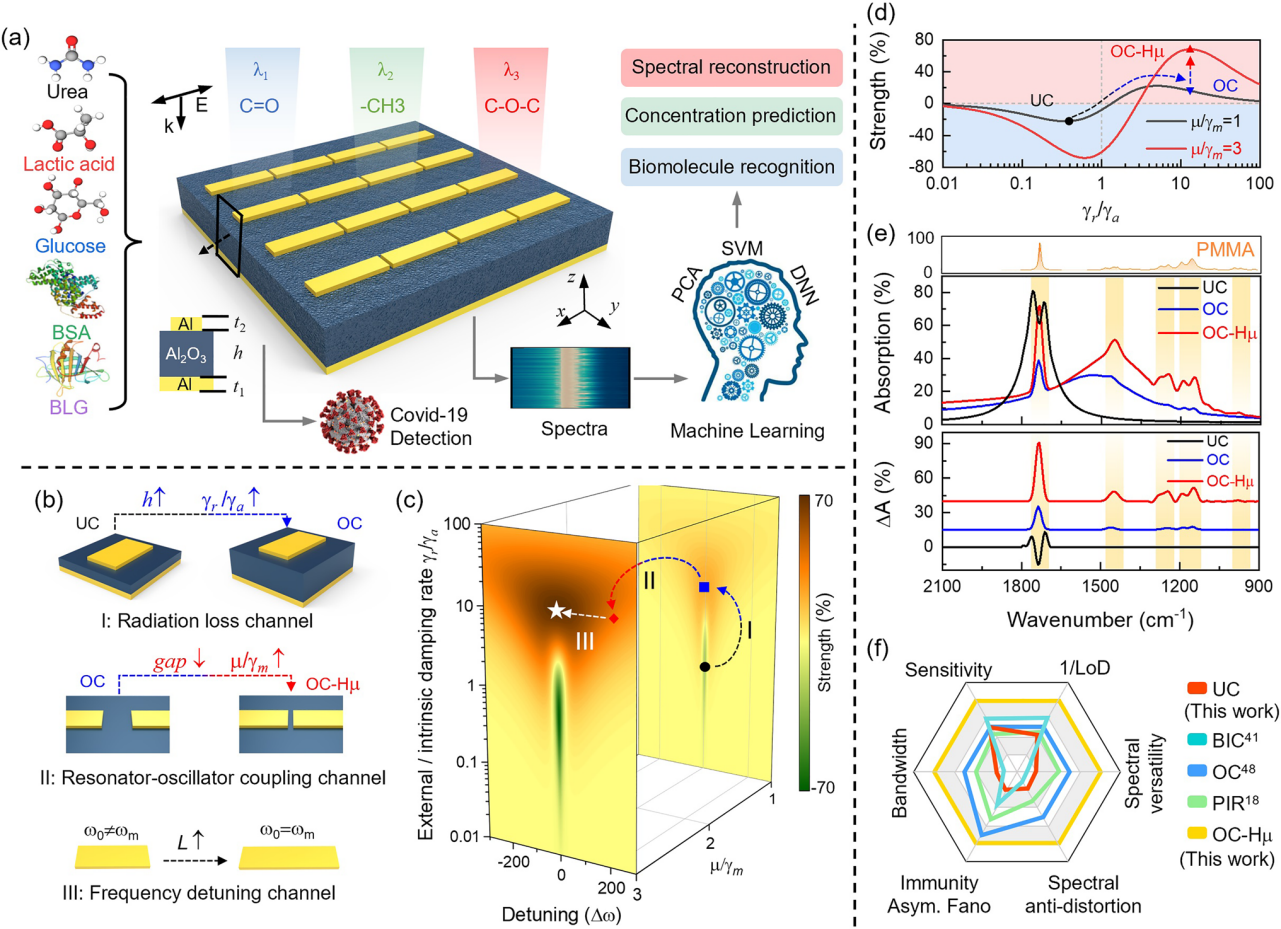
Design principles and main results. **a** Schematic diagram of OC-Hµ for biomolecule sensing. From bottom to top: Al film (*t*_1_ = 100 nm), Al_2_O_3_ (*h* = 550 nm) and Al nanoantenna (*t*_2_ = 60 nm). The gap between adjacent antennas is 40 nm. Five different biomolecules were distributed on the OC-Hµ surface and used to achieve SEIRA spectroscopy. With the assistance of machine learning, we achieved multiple functions such as biomolecule identification, mixture concentration prediction, and spectral reconstruction on a single array. Additionally, we demonstrate the application of the OC-Hµ resonator in the field of COVID-19 detection. **b** Obtained OC-Hµ device by controlling radiation loss channel (I), resonator-oscillator coupling channel (II) and frequency detuning channel (III). First, the device is transformed from UC to OC mode by increasing the thickness of the dielectric layer in MA. This is because the thickness of the dielectric layer can control the ratio of radiative loss (*γ*_*r*_) to absorptive loss (*γ*_*a*_) of the MA. Next, the parameter µ is improved by reducing the gap between adjacent nanoantennas in the OC mode. When µ is greater than the absorption loss of the molecule (*γ*_*m*_), the device changes from the traditional OC to the OC-Hµ mode. The resonant frequency of the MA is modulated by changing the feature size of the nanoantenna. **c** The sensitivity of MA is mapped in three-dimensional space based on TCMT, which is used to intuitively explain the sensitivity adjustment process of the three coupling channels in (**b**). **d** The three-dimensional mapping of sensitivity in (**c**) is reduced to two dimensions (*ω*_*0*_ = *ω*_*m*_) to visually demonstrate the evolution of the device from UC to OC and then to OC-Hµ. **e** The absorption spectra and absorption difference spectra of the above devices coupled with PMMA molecules (the orange shaded area is the fingerprint vibration of PMMA). Among them, UC devices (black) with high *Q* factors can only respond to one fingerprint vibration of PMMA molecules. Traditional OC devices (blue) can simultaneously enhance multiple fingerprint vibrations of PMMA molecules, but the sensitivity is limited. The OC-Hµ device (red) not only enhances multiple fingerprint vibrations of PMMA molecules, but also has ultra-high sensitivity. **f** Performance comparison of OC-Hµ devices with other devices (UC, OC with low µ, BIC and plasma internal reflection (PIR)). For the sake of fairness, all devices have only one resonance mode

The concept of our infrared sensing platform for biomolecule recognition and detection is shown in Fig. 1. As shown in Fig. 1a, the sensing platform is composed of metal-insulating-metal (MIM), also known as MA. The bottom metal of the MA (thickness > 100 nm) blocks all transmission (*T* = 0), allowing the sensing platform to operate in a reflective state. Therefore, the relationship between absorbance (*A*) and reflectance (*R*) is simplified to *A* = 1 – *R*. The material of the top metal is aluminum, which is compatible with the complementary metal–oxide–semiconductor (CMOS) process and is low cost. In addition to being cost-effective, aluminum spontaneously forms a 2–4 nm thick oxide layer (Al_2_O_3_) on its surface to passivate the antenna structure and prevent further oxidation [52]. In addition, the oxide layer supports various modes such as covalent binding and physical adsorption, which provide additional opportunities for biosensing [53]. The top nanoantenna and bottom metal are separated by an Al_2_O_3_ spacer layer. Here, we find based on loss engineering that the sensitivity of MA can be customized by adjusting three channels. The three channels are the radiation loss channel, the resonator-oscillator coupling channel and the frequency detuning channel, respectively. Specifically, in our sensing platform, the radiation loss channel is tuned by the Al_2_O_3_ spacer layer. Increasing the spacer layer thickness increases the ratio of resonator radiation losses to absorption losses, allowing tuning of the resonator from UC (*γ*_*r*_/*γ*_*a*_ < 1) to OC (*γ*_*r*_/*γ*_*a*_ > 1) states (Fig. 1bI, c, d). To stably construct the OC state, we set the spacer layer thickness to 550 nm. The resonator-oscillator coupling channel is related to the molecular absorption loss (*γ*_*m*_) and the coupling coefficient between the resonator-oscillator (µ), that is, *μ*/*γ*_*m*_. We assume that *γ*_*m*_ is constant. Therefore, the resonator-oscillator coupling channel is mainly related to the parameter µ. In general, µ can be tuned by changing the gap between adjacent nanoantennas. A small gap can provide strong near-field enhancement, thereby increasing µ value. In the OC state, we obtain a high µ value OC resonator (OC-Hµ) by reducing the gap between adjacent nanoantennas (the gap is 40 nm) (Fig. 1bII, c, d). Obviously, the increase of µ further improves the sensitivity of the OC resonator (Fig. 1c, d). The frequency detuning channel can be controlled by the feature size and period of the nanoantenna (Fig. 1bIII). By adjusting the frequency detuning channel, maximum sensitivity can be accessed at specific coupling states (Fig. 1cIII). Typically, maximum sensitivity in a specific coupling state occurs when the resonator and oscillator frequencies match (zero detuning) [54]. Therefore, we only show the case of frequency matching in Fig. 1d. The impact of frequency detuning channels on sensitivity will be discussed in detail in the next section.

Through precise control of the above channels, we obtain UC, OC, and OC-Hμ resonators, respectively. A 30 nm thick PMMA was placed on the resonator surface using numerical simulation (Fig. 1e) (See Note S1 for simulation details). The results show that OC-Hμ has excellent sensing performance compared to UC and OC resonators, including higher sensitivity, wider enhancement range, and immune asymmetric Fano resonance (Fig. 1e). Based on these three characteristics, we further expanded the advantages of OC-Hμ resonators in sensing, including lower LOD, spectral versatility and spectral anti-distortion (Fig. 1f). The OC-Hμ resonator combines multiple advantages and surpasses all resonators reported so far, representing a major advancement in the development of SEIRA technology.

### Tailoring Light–Matter Interactions

TCMT is used to understand and tailor light–matter interactions in MA-molecule coupling systems. The absorption spectral dispersion after MA coupling molecules can be calculated by the following equation (see Note S2 for detailed equation derivation) [55],

1 $$A{ = 1} - \left| {\frac{{\kappa^{2} }}{{j(\omega - \omega_{0} ) + (\gamma_{r} + \gamma_{a} ) + \left( {\frac{{\mu^{2} }}{{j\left( {\omega - \omega_{m} } \right) + \gamma_{m} }}} \right)}} - 1} \right|^{2}$$

where ω_0_ and ω_*m*_ are the resonance frequencies of MA and molecular vibration, respectively. *γ*_*r*_ and *γ*_*a*_ represent the radiation and absorption losses of MA, while γ_*m*_ represents the absorption loss of molecules. κ is the coupling coefficient (κ = (2γ_*r*_)^1/2^) between light and MA. µ is the coupling coefficient between MA and the molecule. The enhanced signal spectrum (ΔA) is defined as:

2 $$\Delta A{ = }A - \left. A \right|_{\mu = 0}$$

*A*|_µ=0_ and* A* represent the absorption spectra before and after MA coupling molecules, respectively. We define the sensitivity (*I*_*SEIRA*_) of MA as the intensity of the absorption difference spectrum when *ω* = *ω*_*m*_, that is, *I*_*SEIRA*_ = Δ*A*|_*ω*=*ωm*_ (Fig. S1). According to the above theory, the sensing sensitivity of MA is affected by multiple parameters. To simplify the parameter dimensions, we define the ratio of the loss rate as *f* = *γ*_*r*_/*γ*_*a*_, the molecular coupling parameter as *ξ* = *μ*/*γ*_*m*_, the spectral detuning as *Δω* = *ω*_0_–*ω*_m_.

**Fig. 2:**
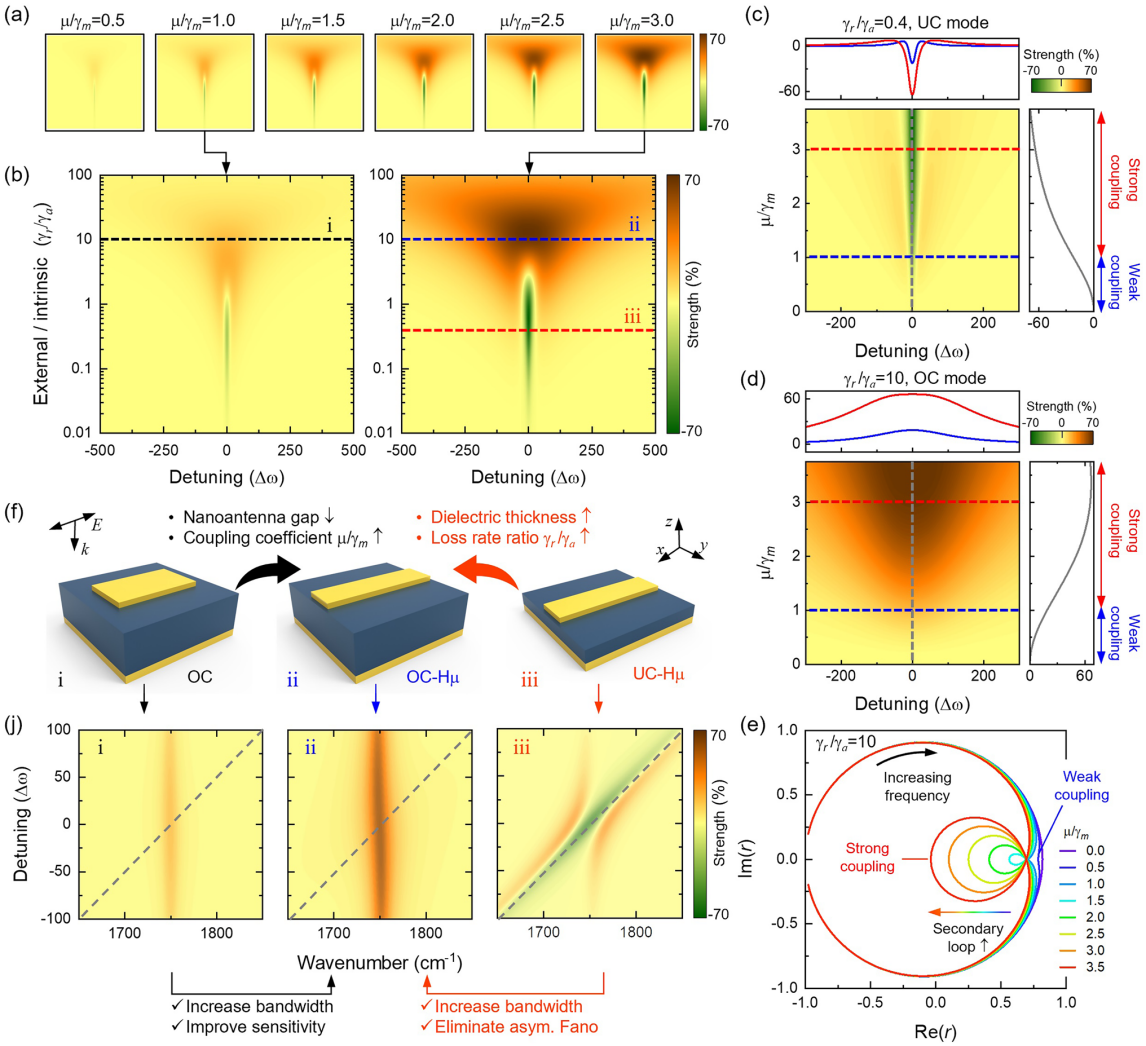
Tailoring light–matter interactions. **a** Calculated 2D mapping of SEIRA enhanced signal based on TCMT as a function of spectral detuning (ω_0_–ω_*m*_) and γ_*r*_/γ_*a*_. Among them, *μ/γ*_*m*_ gradually increases, ranging from 0.5 to 3.0. **b** Zoomed-in 2D mapping of SEIRA sensitivity at *μ/γ*_*m*_ = 1 and *μ/γ*_*m*_ = 3. **c**, **d** To visually demonstrate the influence of *μ/γ*_*m*_ on the SEIRA enhanced signal, the 2D mapping of the SEIRA sensitivity was extracted as a function of spectral detuning and *μ/γ*_*m*_ in UC (γ_*r*_/γ_*a*_ = 0.4) (**c**) and OC (γ_*r*_/γ_*a*_ = 10) (**d**) mode, respectively. Obviously, the sensitivity and bandwidth of the device are significantly enhanced as *μ/γ*_*m*_ increases in OC mode (**d**). **e** Complex representation of Fresnel reflection in OC mode to reveal the sensitivity improvement mechanism. When *µ/γ*_*m*_ > 1, a secondary loop appears in (**e**). The generation of secondary loop indicates that the coupled system changes from weak coupling to strong coupling state. This topological change significantly improves the sensitivity of OC mode. **f** Three types of devices were designed under the framework of TCMT, including OC (i), OC-Hµ (ii) and UC-Hµ (iii). Among them, OC and OC-Hµ devices have the same dielectric layer thickness. OC-Hµ devices can be obtained by optimizing the nanoantenna gap of the OC device. OC-Hµ and UC-Hµ devices have the same nanoantenna gap. The difference is that UC-Hµ has a thinner dielectric layer. **j** Calculated absorption difference spectral mapping for three devices as a function of spectral detuning. The grey dashed line corresponds to the resonant frequency of the device. It is found from (**j**) that the anticrossing behavior disappears when the device transitions from UC to OC mode. The anticrossing behavior is related to the asymmetric Fano resonance [48]. The disappearance of the anticrossing behavior indicates that the OC mode is immune to the interference of the asymmetric Fano resonance. Additionally, bandwidth is significantly enhanced as the device transitions from UC to OC mode. Comparing OC and OC-Hµ, increasing *μ/γ*_*m*_ can further improve the sensitivity and bandwidth of the device. This result is consistent with (**b**)

These three parameters represent the radiation loss channel, the resonator-oscillator coupling channel and the frequency detuning channel, respectively. By adjusting the above three channels, we can customize the sensitivity of SEIRA. For visual presentation, Fig. 2a shows the mapping of sensitivity as Δω and *f* under different *μ/γ*_*m*_ (ranging from 0.5 to 3.0) (see Fig. S2 for detailed mapping of sensitivity). From a macro perspective, the increase in *μ/γ*_*m*_ will further improve the sensing sensitivity of SEIRA. Here, we extract the mapping of sensitivity when *μ/γ*_*m*_ = 1 and *μ/γ*_*m*_ = 3 for detailed analysis (Fig. 2b). Under the modulation of the radiation loss channel, the SEIRA sensitivity is divided into two areas: orange-red (*I*_*SEIRA*_ > 0) and green (*I*_*SEIRA*_ < 0), which form a strong contrast. Generally, *I*_*SEIRA*_ > 0 occurs in OC mode (*γ*_*r*_/*γ*_*a*_ > 1), and *I*_*SEIRA*_ < 0 occurs in UC mode (*γ*_*r*_/*γ*_*a*_ < 1). See Note S2 and Fig. S3 for a detailed explanation of the different sensitivity situations. It is intuitively found that the SEIRA sensitivity has a larger detuned modulation range in the OC mode, indicating that the OC mode can avoid the problem of sensitivity attenuation caused by frequency detuning. This feature of OC mode shows that it can enhance more fingerprint vibrations compared to UC mode. Besides, UC and OC modes exhibit different rules under modulation of the resonator-oscillator coupling channel. For intuitive display, we extract the sensitivity of SEIRA when *γ*_*r*_/*γ*_*a*_ = 0.4 and *γ*_*r*_/*γ*_*a*_ = 10 as the mapping of *μ/γ*_*m*_ and *Δω*. In UC mode (*γ*_*r*_/*γ*_*a*_ = 0.4), the enhancement bandwidth of MA is almost unchanged as *μ/γ*_*m*_ increases (Fig. 2c). In contrast, the enhancement bandwidth in OC mode (*γ*_*r*_/*γ*_*a*_ = 10) increases significantly with the increase of *μ/γ*_*m*_ (Fig. 2d). We attribute the above phenomenon to the large full width at half maximum (FWHM) of the OC mode (Fig. S4). Furthermore, theoretical calculations show that the sensitivity of SEIRA is independent of the Q-factor and absorption intensity of the resonator (Fig. S5). It is worth noting that |*I*_*SEIRA*_| increases with the increase of *μ/γ*_*m*_, whether in UC and OC mode. This is because the increase in *μ/γ*_*m*_ will gradually drive the plasmon-molecule coupling system from a weak coupling to a strong coupling state (Figs. 2e and S6, S7). A distinctive feature of strong coupling is the occurrence of secondary loops in the complex plane [56], corresponding to *μ/γ*_*m*_ > 1. Strong coupling means that we can further enhance the sensitivity in OC mode by adjusting parameter *μ/γ*_*m*_. Frequency detuning channel provide the opportunity to access maximum sensitivity for specific coupling states. The specific coupling state here refers to the situation where *f* and *ξ* are fixed to a certain value. The calculation results in Fig. 2c, d show that the maximum sensitivity occurs when the frequency is matched (*Δω* = 0) regardless of UC or OC mode. It needs to be emphasized that there are two special cases where the maximum sensitivity occurs in the frequency detuning state [47, 48]. These two special cases are beyond the scope of this work. Through numerical calculations, we reveal the dependence of SEIRA sensitivity on the three coupling channels. The analysis results show that the OC mode has a larger bandwidth and can respond to more fingerprint vibrations. In addition, the bandwidth and sensitivity of the OC mode can be further improved by modulating the resonator-oscillator coupling channel.

Modulation of coupling channels provides guidance for tailoring light–matter interactions and designing devices with maximum sensitivity. Under the theoretical framework (Fig. 2b), we designed MAs with different parameters. First, we increase *γ*_*r*_/*γ*_*a*_ by increasing the thickness of the dielectric layer and reducing the unit cell period to obtain an OC resonator (Fig. 2fi, see Fig. S8 for OC design details). Next is to increase *μ/γ*_*m*_ in OC mode. There are two ways to improve *μ/γ*_*m*_, which are increasing the number of molecules (Fig. S9) and reducing the gap between adjacent nanoantennas (Fig. S10). Considering that the original intention of SEIRA technology is to detect small amounts of molecules or ultra-thin film systems. Therefore, here we use reducing the gap between adjacent nanoantennas to improve *μ/γ*_*m*_, and the corresponding device is called OC-Hµ resonator (Fig. 2fii). For comparison, we obtained a device with high µ in UC mode by reducing the gap between adjacent nanoantennas, called UC-Hµ (Fig. 2fiii). The main difference from OC-Hµ is that the dielectric layer of UC-Hµ is thinner and *γ*_*r*_/*γ*_*a*_ < 1. The calculation results show that increasing *μ/γ*_*m*_ can improve SEIRA sensitivity and sensing bandwidth in OC mode (Fig. 2ji, jii). Comparing OC-Hµ and UC-Hµ, increasing *γ*_*r*_/*γ*_*a*_ can significantly increase the sensing bandwidth (Fig. 2b). Importantly, the anticrossing behavior in the absorption difference spectra disappears when the resonator transitions from UC-Hµ to OC-Hµ mode, indicating that OC-Hµ can eliminate the asymmetric Fano resonance (Fig. 2jii, jiii). The elimination of asymmetric Fano resonance allows us to directly obtain the intrinsic fingerprint vibration information of molecules without the need for secondary correction. We calculated the derivative spectra of the absorption difference spectra and further discussed the significant advantages brought by the elimination of asymmetric Fano resonances (Fig. S11). The results show that the derivative spectrum obtained based on the OC-Hµ resonator remains consistent with the intrinsic derivative spectrum of the molecule. This feature makes the OC-Hµ resonator uniquely potential in analyzing protein secondary structure.

### Sensing Characterization of OC-Hμ

**Fig. 3:**
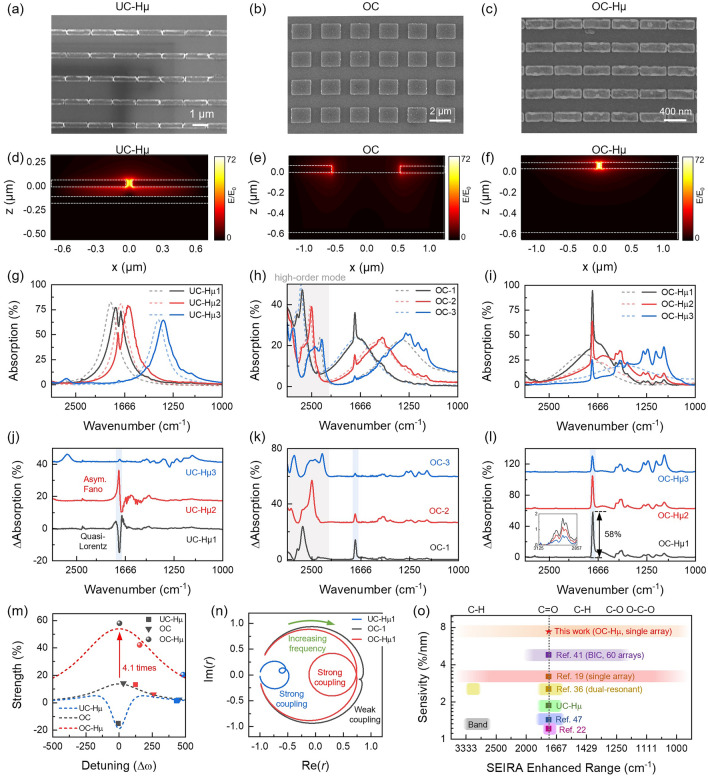
Experiment characterization of OC-Hµ sensing performance. **a–c** SEM images of UC-Hµ (**a**), OC (**b**), and OC-Hµ (**c**) devices are shown respectively. **d–f** Simulated the electric field distribution of UC-Hµ (**d**), OC (**e**), and OC-Hµ (**f**) devices. **g–i** Measured the absorption spectra of UC-Hµ (**g**), OC (**h**), and OC-Hµ (**i**) devices with (solid curves) and without (bashed curves) 8 nm PMMA. In each mode, similar resonant frequencies are obtained by customizing the unit cell period and the structure of the nanoantenna. The spectra of different resonant frequencies in a particular mode are represented in black, red, and blue, respectively. The resonant peaks of UC-Hµ (**g**) and OC-Hµ (**i**) are clean in the mid-infrared range. There are high-order modes in OC devices in the range of 2000–3333 cm^−1^ (grey shaded area in (**h**)). **j–l** Extracted the SEIRA enhanced spectra from (**g**–**i**), respectively. The blue shaded area corresponds to the C=O vibration of PMMA, and the resonant frequency is 1730 cm^−1^. The illustration in (**l**) shows the vibration mode of the C–H bond in PMMA in the range of 2857–3125 cm^−1^. **m** SEIRA signal intensity at C=O bonds (1730 cm^−1^, points) as a function of spectral detuning. The dashed curves represent the theoretical fit (see Equation (S8) for the fitting formula). **n** The complex representation of Fresnel reflection of UC-Hµ1 (blue), OC-1 (black) and OC-Hµ1 (red) devices was inverted using TCMT. Other inversion results are shown in Fig. S14 and Table S3. There is no secondary loop in OC-1 because µ/γ_*m*_ < 1. In this case, the system is in a weak coupling state. In UC-Hµ and OC-Hµ, secondary loops appear in the curves because µ/γ_*m*_ > 1. In this case, the system is strong coupling state. **o** Comparison between this work and previous work

To experimentally verify the above ideas, we prepared UC-Hµ (Fig. 3a), OC (Fig. 3b) and OC-Hµ (Fig. 3c) devices, respectively (see Note S3 for fabrication details). Among them, the UC-Hµ devices consist of a 100 nm metal aluminum film, a 100 nm thick dielectric layer and a 60 nm thick aluminum nanoantenna (Fig. 3d). The gap between adjacent nanoantennas is 40 nm, which is used to increase the coupling between the naoantennas and molecules. The OC devices consist of a 100 nm metal aluminum film, a 550 nm thick dielectric layer and a 60 nm thick aluminum nanoantenna (adjacent nanoantennas maintain a relatively large gap) (Fig. 3e). Based on the OC device, µ is improved by reducing the gap (40 nm) between adjacent nanoantennas (Fig. 3f). The results of electric field simulation (Fig. 3d-f) show that the small gap between adjacent nanoantennas can not only increase the electric field intensity, but also strongly constrain the electric field between the gaps, thereby increasing the value of µ. The resonant frequencies of all devices were tailored to specific wavelength bands by adjusting the unit cell period and nanoantenna structure size (see Table S1 for structure size). It should be emphasized that the spectral vibration of UC-Hµ and OC-Hµ devices in the mid-infrared region is clean and has no interference from additional vibration modes. This is particularly attractive because it allows highly spectrally selective enhancement of spectrally rich molecular fingerprint information [20].

Next, 8 nm thick PMMA was used as a probe molecule to characterize the SEIRA performance of different devices (see Fig. S12 for PMMA thickness characterization). The absorption spectra of the device before (dashed curves) and after (solid curves) coupling molecules are shown in Fig. 3g-i. It should be noted that the loading of molecules causes the resonance frequency of the device to red-shift. For clarity, we define the resonant frequency of the device without coupled molecules as *ω*_0_, and the resonant frequency of the device with coupled molecules as *ω*_01_. Since this work focuses on the system after the device is coupled to the molecule, the detuning in the experiment is defined as the difference between *ω*_m_ and *ω*_01_. To obtain the SEIRA spectra of the molecules, we used asymmetric least squares smoothing (AsLSS) algorithm to baseline calibrate the coupled spectra. The extracted absorption difference spectra are shown in Fig. 3j-l. Due to the small FWHM of UC-Hµ, it cannot sense fingerprint vibrations far away from the resonance peak. In addition, the absorption difference spectra of UC-Hµ presents two perturbation lineshapes, namely quasi-Lorentz lineshape (*ω*_01_ = *ω*_*m*_, black curve in Fig. 3j) and asymmetric Fano lineshape (*ω*_01_ ≠ *ω*_*m*_, red curve in Fig. 3j) [57]. These two lineshapes do not match the Lorentz lineshape of the molecule and require secondary correction to obtain the intrinsic vibration information (resonant frequency, bandwidth, and intensity) of the molecule. In contrast, the OC mode has the advantages of broadband enhancement and immune asymmetric Fano resonance (Fig. 3k). However, the traditional OC mode has limited sensitivity and enhancement bandwidth due to the lower µ. In addition, the high-order modes of the traditional OC mode at short wavelengths (2000–3333 cm^−1^) hinder the acquisition of molecular fingerprint information in this band. These shortcomings of the traditional OC model can be solved by increasing the value of µ. As shown in Fig. 3l, OC-Hµ has ultra-high SEIRA sensitivity compared to UC-Hµ and OC modes. In particular, the enhancement of C=O bonds by OC-Hµ reaches an astonishing 58% when the spectra are matched (*ω*_01_ = *ω*_*m*_, Fig. 3l). Since the film thickness of PMMA is 8 nm, the sensitivity of OC-Hµ is 7.25% nm^−1^ (58%/8 nm = 7.25% nm^−1^). Compared with the traditional OC resonators, the sensitivity of OC-Hµ is improved by 4.1 times (Fig. 3m). In addition, OC-Hµ’s enhanced bandwidth covers the entire mid-infrared (1000–3333 cm^−1^ or 3–10 µm), which can detect all infrared fingerprint vibrations of PMMA (Fig. 3l). Even at the edge of the bandwidth, OC-Hµ still has good excitation and detection capabilities (see the inset of Fig. 3l). To the best of our knowledge, both the sensitivity (7.25% nm^−1^) and enhancement bandwidth (7 µm or 2333 cm^−1^) of OC-Hµ are the largest in SEIRA to date (Fig. 3o, Table S2). Not only that, OC-Hµ can also eliminate the interference of asymmetric Fano resonance, so it can directly obtain a spectral lineshape consistent with the molecular fingerprint vibration spectrum. Next, we inverted the complex representation of Fresnel reflection based on TCMT (Fig. 3n, see Fig. S13 and Table S3 for TCMT fitting results). Consistent with Fig. 2e, reducing the gap between adjacent nanoantennas induces the generation of secondary loops. This topological change indicates that the coupling between the optical microcavity and the molecules changes from weak coupling to strong coupling [56, 58], thereby achieving stronger SEIRA sensitivity.

### Spectral Versatility and Low Detection Limits

**Fig. 4:**
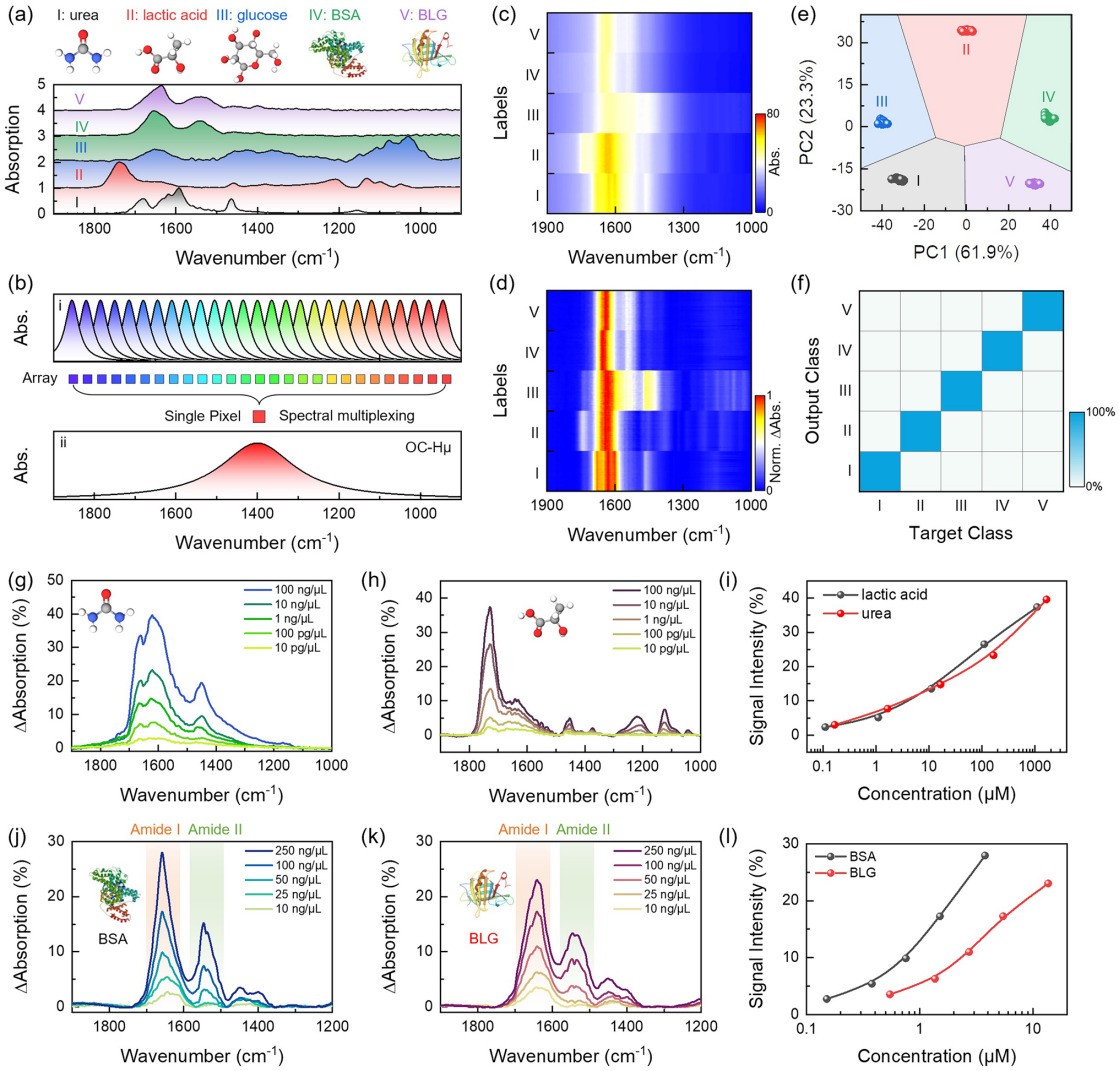
Spectral versatility and low detection limit characteristics of OC-Hµ devices. **a** Fingerprint absorption spectra of five molecules. The five types of molecules are urea, lactic acid, glucose, BSA, BLG. **b** (i) The multi-array method is used to achieve spectral versatility because the FWHM of traditional device is small. Among them, each array maps a resonant frequency. (ii) Since OC-Hµ has a large FWHM, spectral versatility can be achieved through a single array. **c** Measured the absorption spectra of OC-Hµ devices when coated with different molecules. Among them, there are 50 groups of absorption spectra for each molecule. **d** Extracted the normalized absorption difference spectrum from (**c**). The results show that spectral versatility can be achieved using a single array of OC-Hµ devices. Importantly, the fingerprint vibration is still significantly enhanced even far away from the plasmon resonance peak. **e** The weight of each spectral fraction in 2D space after PCA of the measured spectra in (**c**). Each cluster represents a type of molecule. Since each molecule does not overlap spatially, we can identify different molecular species. **f** SVM classification accuracy for different molecules. The confusion plot shows that the accuracy of molecular identification is 100%. **g**, **h** Absorption difference spectra of OC-Hμ loaded with different urea concentrations (**g**) and different lactic acid concentrations (**h**). **i** Extracted the maximum SEIRA intensity as a function of molecule concentration from (**g**) and (**h**). **j**, **k** Absorption difference spectra of OC-Hμ loaded with different BSA concentrations (**j**) and different BLG concentrations (**k**). **l** Extracted SEIRA intensity of Amide I as a function of protein concentration

The ultra-bandwidth, ultra-sensitivity and immune asymmetric Fano resonance of OC-Hµ extend the three characteristics of spectral versatility, low LOD and spectral anti-distortion respectively. We randomly selected 5 biomolecules (urea, lactic acid, glucose, BSA, BLG) to demonstrate the spectral versatility of OC-Hµ. It is known that different molecules have different fingerprint vibrations (Fig. 4a). Traditional nanoantenna designs cannot match multiple fingerprint vibrations simultaneously due to their limited bandwidth. To solve this problem, nanoantennas covering different wavelength bands need to be customized to achieve spectral versatility (Fig. 4bi) [20]. Although this method can increase bandwidth, it also faces more structural design and larger device size. The ultra-bandwidth characteristics of OC-Hµ provide a new way to achieve spectral versatility using a single nanoantenna array (array size is 100 µm × 100 µm). Here, we loaded 5 biomolecules onto the surface of the OC-Hµ device sequentially (see Sect. 2.1 for experimental details), and using FTIR to collect absorption spectra (Fig. 4c). The extracted absorption difference spectra show that the OC-Hµ device can achieve spectral versatility even with a single-array (Fig. 4d). In addition, fingerprint vibrations even far away from the plasma resonance frequency are significantly enhanced (Fig. 4d). Based on the rich fingerprint vibration information obtained by OC-Hµ, we used PCA and SVM to achieve biomolecule classification (Figs. 4e and S15) and fingerprint retrieval (accuracy is 100%) (Fig. 4f). It should be emphasized that the ultra-broadband characteristics of OC-Hµ have the potential to detect more analytes.

Next, we demonstrated the lower LOD of the OC-Hμ resonator. First, we loaded urea and lactic acid solutions with different concentrations onto the OC-Hμ device surface (see Sect. 2.1 for experimental details). The absorption difference spectra of different concentrations of urea and lactic acid are shown in Fig. 4g, h (the measurement results of glucose are shown in Fig. S16). Obviously, the intensity of the absorption difference spectrum is positively correlated with the molecular concentration (Fig. 4i). Relying on this correlation, we can achieve quantitative detection of molecules. It is worth noting that even at a molecule concentration of 10 pg μL^−1^, a signal contrast of approximately 3% was still observed from the absorption difference spectrum. This means we can detect small molecules at low concentrations. Further, we loaded different concentrations of BSA and BLG onto the OC-Hμ surface. It should be emphasized that the loading concentration ranges for proteins and small molecules (lactic acid and urea) are different due to their different molar masses. Figure 4j, k respectively extracted the absorption difference spectra of proteins with different concentrations. For both proteins, the vibration intensity of Amide I and Amide II increases with increasing protein concentration. Even at a concentration of 10 ng μL^−1^, a signal contrast of 2.5% was still observed. We further plotted the absorption intensity of Amide I as a function of protein concentration. 3σ of noise was used to evaluate the LOD of OC-Hμ. The LOD of BSA is 63 nM, and the LOD of BLG is 186 nM. This LOD corresponds to proteins on the array down to zeptomole magnitude. In addition, we evaluated the signal enhancement at different wavelengths (Fig. S17, Table S4). The results show that the signal enhancement at different wavelengths satisfies the Langmuir EXT1 formula. Therefore, we can achieve quantitative analysis at different wavelengths.

### Spectral Anti-Distortion

**Fig. 5:**
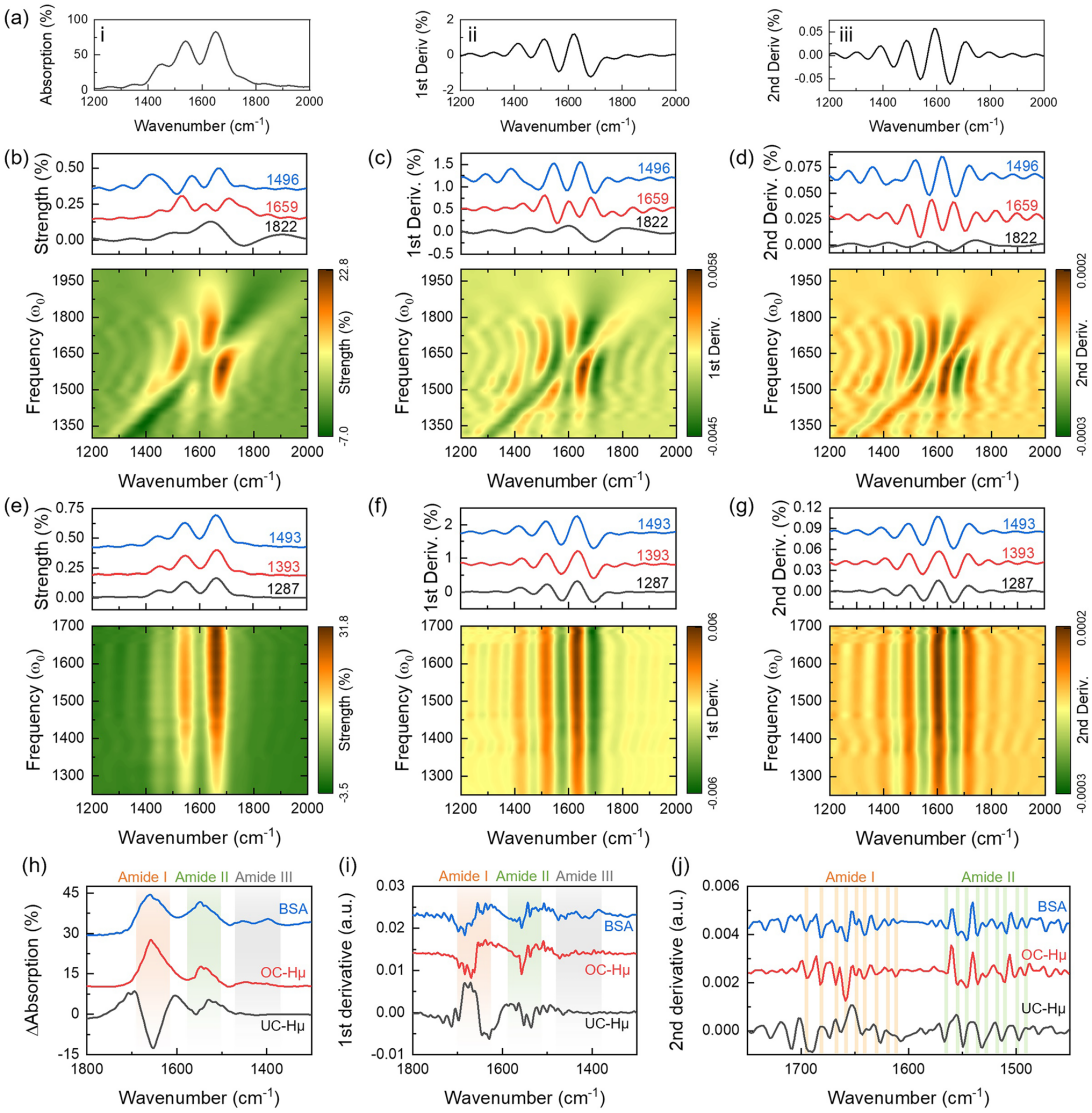
Spectral anti-distortion characteristic of OC-Hµ devices. **a** (i) Simulated the absorption spectrum of a 1 µm thick protein film on a gold surface. Calculated the first derivative spectra (ii) and second derivative spectra (iii) from (ii). **b** Simulated absorption difference spectra mapping of UC-Hµ devices loaded with proteins as a function of spectral detuning. **c**, **d** Calculated the first derivative spectra (**c**) and second derivative spectra (**d**) from (b). **e** Simulated absorption difference spectra mapping of OC-Hµ devices loaded with proteins as a function of spectral detuning. **f**, **g** Calculated the first derivative spectra (**f**) and second derivative spectra (**g**) from (**e**). **h** Comparison of absorption difference spectra of OC-Hμ (red) and UC-Hμ (black). The blue curve is the absorption spectrum of pure BSA protein on a gold mirror. **i**, **j** Calculated the first (**i**) and second derivatives (**j**) from (**h**). Since the OC-Hμ device has the characteristics of broadband enhancement and immunity to asymmetric Fano resonance, spectra and derivative spectra consistent with molecular fingerprints can be directly obtained

Next, we demonstrated the spectral anti-distortion properties of the OC-Hµ resonator. First, we use numerical simulation methods to analyze and discuss. In the simulation, protein is used as a probe molecule, and the absorption spectrum and derivative spectrum are shown in Fig. 5a (the complex permittivity function is taken from Durmaz et al. [59]). The UC-Hµ device is used for comparison and its dimensional parameters are shown in Fig. S18a. A 10 nm thick protein film was placed on the surface of the UC-Hµ device and absorption spectra (Fig. S18c) were collected using a frequency domain monitor. Further, the absorption difference spectra was extracted from the absorption spectra, as shown in Fig. 5b. From the absorption difference spectra, we observed obvious anticrossing phenomenon and asymmetric Fano lineshapes. It should be emphasized that the asymmetric Fano lineshapes change with the change of the UC-Hµ resonant frequency. Affected by this, it is difficult to obtain the true vibrational information of the protein from the absorption difference spectra. In addition, the asymmetric Fano lineshapes also seriously affects the first derivative spectra (Fig. 5c) and the second derivative spectra (Fig. 5d). As a result, the UC-Hµ device has challenges in resolving the secondary structure of protein molecules. In contrast, OC-Hµ device is immune to the effects of asymmetric Fano lineshapes. Based on this characteristic of OC-Hµ, we can directly obtain the fingerprint vibration information of the protein from the absorption difference spectra (Fig. 5e). The parameter settings and absorption spectra of OC-Hµ are shown in Fig. S18. In addition, for OC-Hµ, the absorption difference spectra always maintains a spectral lineshape consistent with the protein fingerprint vibration regardless of how the OC-Hµ resonant frequency changes. Not only that, the first derivative spectra (Fig. 5f) and the second derivative spectra (Fig. 5g) obtained based on the OC-Hµ device are also consistent with the derivative spectra of the protein (Fig. 5a). We summarize this as the spectral anti-distortion properties of OC-Hµ. Based on this property, OC-Hμ devices provide the opportunity to accurately resolve protein secondary structures.

Next, we experimentally demonstrated the advantages of OC-Hµ in resolving protein secondary structures. We extracted the absorption difference spectra of BSA on the OC-Hμ and UC-Hμ devices respectively, as shown in Fig. 5h. The BSA fingerprint absorption spectrum at 500 ng μL^−1^ was used for comparison (blue curve in Fig. 5h). The first derivative (Fig. 5i) and second derivative (Fig. 5j) of the BSA absorption spectrum were calculated, respectively. Consistent with the numerical simulation, regardless of the absorption difference spectrum or the derivative spectrum, the measurement results of OC-Hμ are consistent with the spectrum of pure BSA. Therefore, compared with UC-Hμ, the OC-Hμ device can obtain protein fingerprint vibration information and secondary structure without secondary calibration. This feature of the OC-Hμ device represents a major advance for SEIRA in obtaining vibrational information on molecular fingerprints. It should be emphasized that this characteristic of OC-Hμ also makes SEIRA spectroscopy comparable to the relatively mature surface-enhanced Raman spectroscopy, that is, the complex vibrational information of the molecule can be recovered only through baseline correction.

### Mixture Classification, Concentration Prediction, and Spectral Reconstruction

**Fig. 6:**
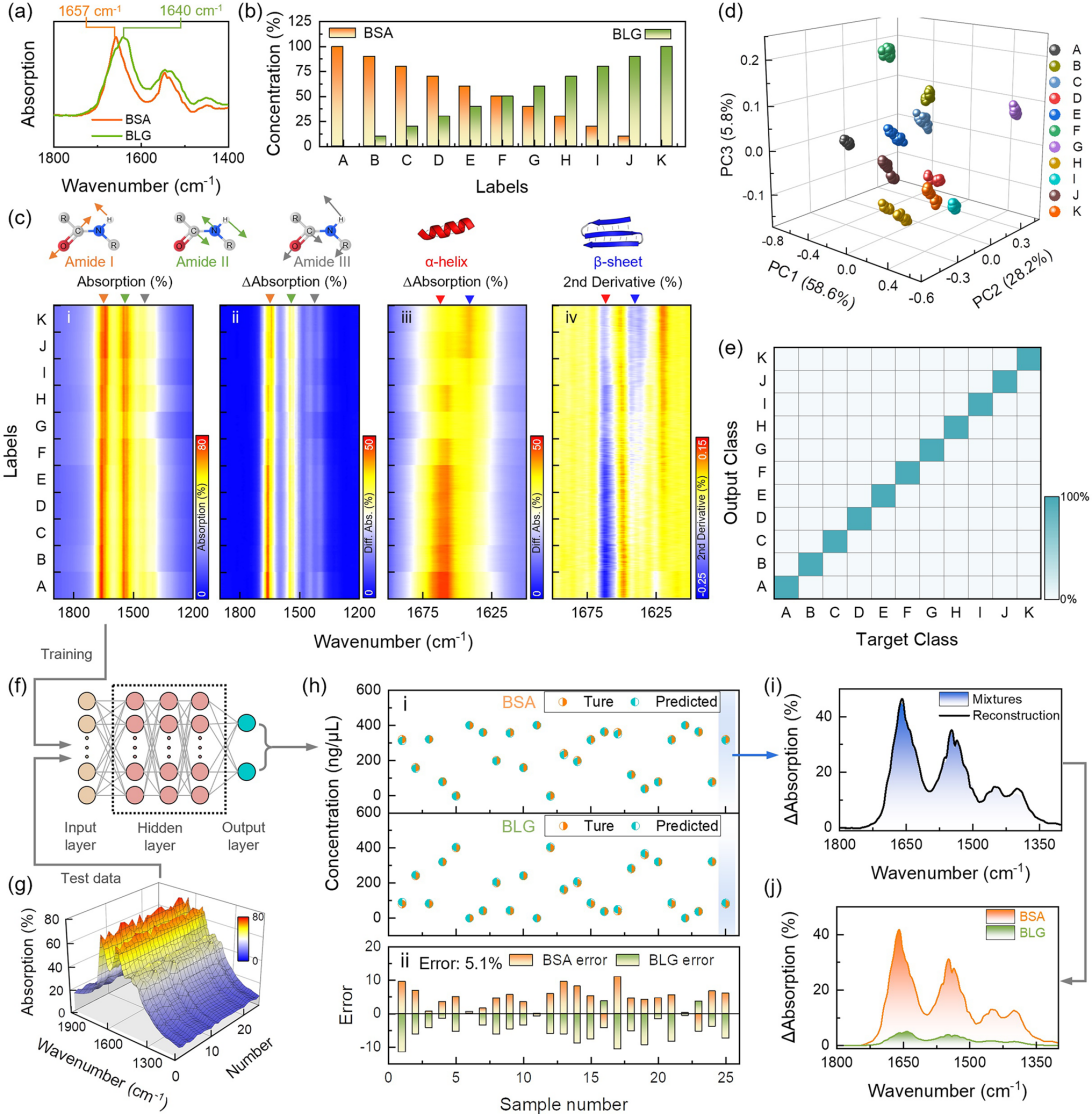
Application of OC-Hµ devices in mixture classification, concentration prediction and spectral reconstruction. **a** Differences in absorption spectra of BSA and BLG proteins. **b** The bar plot of the titrated concentration mixes of BSA and BLG. **c** Collected absorption spectra and data analysis at different protein mixing ratios. (i) is the absorption spectra. (ii, iii) Extracted absorption difference spectra from (i). (iv) Calculated second derivative from (ii). **d** The weight of each spectral fraction in 3D space after PCA on the measured spectra. Each cluster represents a mixing ratio. **e** SVM was used to classify the measured spectra. The confusion plot shows that the accuracy of identification of mixture molecules is 100%. **f** MM-DNN model for mixture concentration prediction. **g** A new set of mixed spectra of BSA and BLG was measured, which was used as a test set for concentration prediction. **h** Concentration prediction results. (i) The true and predicted concentrations of BSA and BLG. (ii) Average error. **i** Spectral reconstruction results of sample 25. Blue shading: absorption difference spectrum of mixture proteins. Black curve: reconstructed mixed spectrum. **j** Decompose the mixed spectrum in (**i**) into a single spectrum of BSA and BLG

Next, we investigated the potential of the OC-Hµ device for mixture classification, concentration prediction, and spectral reconstruction. The subtle differences present in the Amide I band between BSA and BLG provide feasibility for mixture classification (Fig. 6a). This difference arises from the different folding patterns of BSA and BLG on Amide I. Here, we designed sample sets of different mixtures of titrated percentage combinations of BSA and BLG (Fig. 6b). The absorption spectra of these different sample sets were measured using the OC-Hµ device (Fig. 6ci). Each sample set contains 50 sets of spectra, containing a total of nearly 560,000 data points. The large amount of data provides an ideal opportunity to use ML models. In the absorption difference spectra, three main vibration peaks of the protein: Amide 1, Amide II, and Amide III were observed (Fig. 6cii). By observing the vibration mode of Amide I (Fig. 6ciii), it was found that the fingerprint vibration corresponding to 1657 cm^−1^ gradually weakened as the proportion of BSA in the mixture decreased. We attribute this to differences in protein conformation. Here, we calculated the second derivative of Amide I and performed conformational analysis (Fig. 6civ). The secondary structure of the Amide I band shows that there are mainly *α*-helices in the BSA protein, and the BLG protein contains α-helices and β-sheets.

Due to the reduction properties of OC-Hµ on fingerprint vibration spectra, we can achieve mixture analysis by analyzing the secondary structure of the Amide I band. However, this method is not necessarily optimal in terms of accuracy of identification and time required to obtain an answer. Therefore, we introduced ML for mixture analysis. The measurement data sets in Fig. 6ci are imported into PCA and SVM respectively for mixture classification and identification. By analyzing the distribution of measurement data in 3D space, we can identify different mixing ratios (Figs. 6d and S19). Furthermore, with the assistance of SVM, we achieved 100% mixture identification (Fig. 6e). Further, we constructed a regression model based on multimodal deep neural network (MM-DNN) for concentration prediction and spectral reconstruction of mixtures (Fig. 6f, see Sect. 2.3 for MM-DNN model details). The data set in Fig. 6ci is used to train the MM-DNN model, and the newly obtained measured spectral data (Fig. 6g) is input into the trained MM-DNN model as a test set. The concentration prediction results are very close to the measured concentrations (Fig. 6hi), with an accuracy of 96.8% (Fig. S20) and an average error of 5.1% (Fig. 6hii). Furthermore, spectral reconstruction can be achieved using the predicted concentration ratio of the mixture, the spectrum of the mixture, and the predefined spectra of BSA and BLG. Taking sample 25 as an example, the concentration ratio of BSA and BLG is 4:1. Through spectral reconstruction, the total overlapping spectra of the heterogeneous sample (Fig. 6i) can be de-overlapped into separate spectra of BSA and BLG (Fig. 6j). Finally, we use MSE to evaluate the accuracy of spectral reconstruction. Generally, the smaller the MSE, the higher the reconstruction accuracy. For sample 25, the calculated MSE is 0.007%. The low reconstruction error indicates a high level of separation accuracy.

### SARS-CoV-2 Detection

**Fig. 7:**
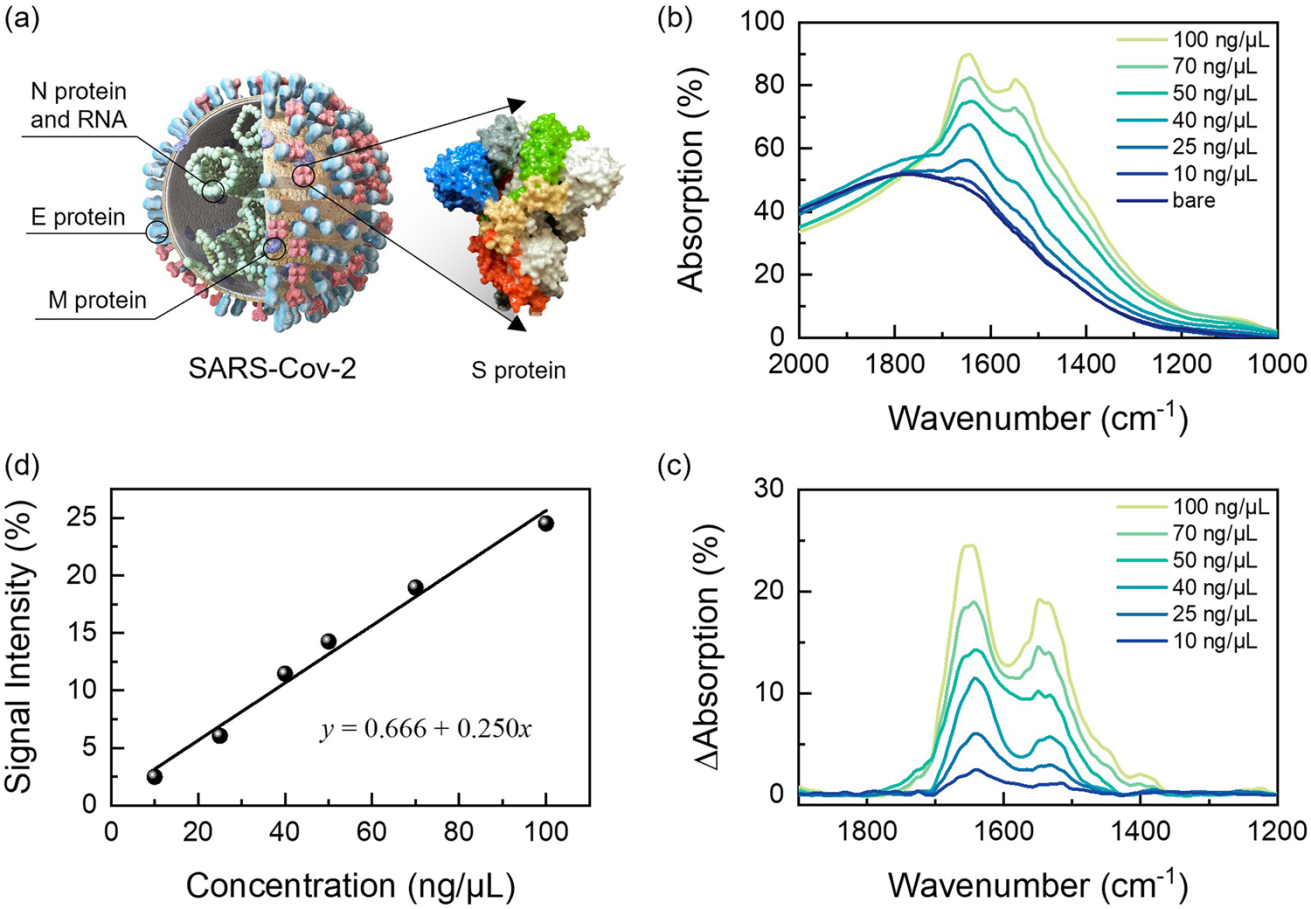
Demonstration of SARS-CoV-2 detection using OC-Hµ device. **a** Schematic diagram of the SARS-CoV-2 virus. The inset is a schematic diagram of the spike protein. **b** Measured absorption spectra when different concentrations of spike protein were loaded onto the OC-Hµ device surface. **c** Extracted the absorption difference spectra from (**b**) using AsLSS. **d** Extracted vibrational intensity of Amide I as a function of spike protein concentration. The solid line is the fitting curve

In 2019, the global outbreak of novel coronavirus pneumonia posed a significant threat to human life worldwide [60]. Despite considerable efforts in vaccination, prevention, and treatment, the shortage of healthcare resources presented substantial challenges for low- and middle-income countries [61]. While the era of COVID-19 pandemic has largely subsided, the exploration of SARS-CoV-2 detection technologies continues unabated. The development of SARS-CoV-2 detection technology is important for containing the outbreak of future new pandemics [62–65]. In this study, we explore the potential of OC-Hµ devices in the field of SARS-CoV-2 detection. COVID-19 is caused by a novel beta coronavirus, comprising a single-stranded positive RNA genome and four structural proteins (spike protein (S), envelope protein (E), matrix protein (M), and nucleocapsid protein (N)) (Fig. 7a) [66]. The mechanism of novel coronavirus infection involves the interaction of the spike protein with the angiotensin-converting enzyme 2 receptor, facilitating cellular entry and causing pneumonia. Therefore, the detection of the spike protein can facilitate the diagnosis of SARS-CoV-2 or the development of vaccines. Here, we loaded solutions containing different concentrations of the spike protein onto the surface of OC-Hµ and collected absorption spectra using FTIR (Fig. 7b). From the absorption difference spectra, we observed two distinct fingerprint vibration modes, representing Amide I and Amide II vibrations (Fig. 7c). Subsequently, we extracted the vibration intensity of Amide I as a function of spike protein concentration (Fig. 7d). The results demonstrated a linear correlation between the vibration intensity of Amide I and the concentration of the spike protein. Even at the lowest concentration of 10 ng μL^−1^, the OC-Hµ device detected approximately 3.2% signal contrast. These findings indicate the suitability of OC-Hµ for the detection of novel coronaviruses.

## Conclusions

In summary, we achieved an OC-Hµ device with high-performance SEIRA effect by precisely controlling the radiation loss channel, resonator-oscillator coupling channel and frequency detuning channel. Among them, the radiation loss channel is used to convert UC devices into OC devices. Resonator-oscillator coupling channel is used to transform OC devices from weak coupling to strong coupling. Frequency detuning channel is used to match target fingerprints. The OC-Hµ device obtained based on the above strategy has strong light–matter interaction. Using 8 nm thick PMMA as the probe molecule, OC-Hµ simultaneously achieves ultra-broadband (3–10 µm) and ultra-sensitive (7.25% nm^−1^) fingerprint detection. To the best of our knowledge, this enhancement is the largest reported among existing SEIRA technologies. The ultra-high sensitivity enables OC-Hµ to have a detection limit down to the zeptomoles level. In addition, compared with UC devices, OC-Hµ is immune to asymmetric Fano resonance. The synergy of immune asymmetric Fano resonance and ultra-broadband makes it possible to achieve fingerprint retrieval on a single array (array size is 100 × 100 µm^2^). As a demonstration, all fingerprint vibration information of five biomolecules was obtained based on the OC-Hµ device. The rich vibrational information of fingerprints provides opportunities to employ ML for species classification and identification. It should be emphasized that the OC-Hµ platform has broad applicability and can detect almost all molecules with infrared fingerprint vibrations. At the same time, the enhanced spectrum obtained based on OC-Hµ can be directly compatible with existing infrared spectral libraries, which is attributed to the anti-spectral distortion characteristics of the OC-Hµ device. This characteristic of OC-Hµ also gives it a unique advantage in analyzing protein secondary structure. In addition, we demonstrated multiple applications of the OC-Hµ device, including mixture classification, concentration prediction, and spectral reconstruction. Thanks to the many properties of OC-Hµ, 100% mixture identification and spectral reconstruction can be achieved even for protein molecules with highly overlapping spectra. This shows that OC-Hµ can easily identify various mixtures. Finally, we verified that the OC-Hµ device can be used for SARS-CoV-2 virus detection. We believe that the enhanced coupling strategy we demonstrated and the proposed OC-Hµ resonator will drive significant advances in SEIRA technology, while also providing new insights into other fields, including enhanced spectroscopy techniques [67], waveguide sensing platform [68–70], quantum photonics [8], optical computing [71, 72], and the study of light–matter interactions [73].

## Supplementary Information

Below is the link to the electronic supplementary material.

Supplementary file1 (DOCX 25350 kb)

## References

[1] T. Weber, L. Kühner, L. Sortino, A. BenMhenni, N.P. Wilson et al., Intrinsic strong light–matter coupling with self-hybridized bound states in the continuum in van der Waals metasurfaces. Nat. Mater. **22**, 970–976 (2023). 10.1038/s41563-023-01580-7 37349392 10.1038/s41563-023-01580-7 PMC10390334

[2] A.H. Dorrah, F. Capasso, Tunable structured light with flat optics. Science **376**, eabi6860 (2022). 10.1126/science.abi6860 35446661 10.1126/science.abi6860

[3] F. Neubrech, C. Huck, K. Weber, A. Pucci, H. Giessen, Surface-enhanced infrared spectroscopy using resonant nanoantennas. Chem. Rev. **117**, 5110–5145 (2017). 10.1021/acs.chemrev.6b00743 28358482 10.1021/acs.chemrev.6b00743

[4] C. Xu, Z. Ren, H. Zhou, J. Zhou, C.P. Ho et al., Expanding chiral metamaterials for retrieving fingerprints via vibrational circular dichroism. Light Sci. Appl. **12**, 154 (2023). 10.1038/s41377-023-01186-3 37357238 10.1038/s41377-023-01186-3 PMC10290984

[5] C. Xu, Z. Ren, H. Zhou, J. Zhou, D. Li et al., Near-field coupling induced less chiral responses in chiral metamaterials for surface-enhanced vibrational circular dichroism. Adv. Funct. Mater. **34**, 2314482 (2024). 10.1002/adfm.202314482

[6] G. Li, S. Zhang, T. Zentgraf, Nonlinear photonic metasurfaces. Nat. Rev. Mater. **2**, 17010 (2017). 10.1038/natrevmats.2017.10

[7] U. Aslam, V.G. Rao, S. Chavez, S. Linic, Catalytic conversion of solar to chemical energy on plasmonic metal nanostructures. Nat. Catal. **1**, 656–665 (2018). 10.1038/s41929-018-0138-x

[8] T. Santiago-Cruz, S.D. Gennaro, O. Mitrofanov, S. Addamane, J. Reno et al., Resonant metasurfaces for generating complex quantum states. Science **377**, 991–995 (2022). 10.1126/science.abq8684 36007052 10.1126/science.abq8684

[9] W. Wang, M. Ramezani, A.I. Väkeväinen, P. Törmä, J.G. Rivas et al., The rich photonic world of plasmonic nanoparticle arrays. Mater. Today **21**, 303–314 (2018). 10.1016/j.mattod.2017.09.002

[10] J. Kozuch, K. Ataka, J. Heberle, Surface-enhanced infrared absorption spectroscopy. Nat. Rev. Meth. Primers **3**, 70 (2023). 10.1038/s43586-023-00253-8

[11] D. Li, C. Xu, J. Xie, C. Lee, Research progress in surface-enhanced infrared absorption spectroscopy: from performance optimization, sensing applications, to system integration. Nanomaterials **13**, 2377 (2023). 10.3390/nano13162377 37630962 10.3390/nano13162377 PMC10458771

[12] D. Dregely, F. Neubrech, H. Duan, R. Vogelgesang, H. Giessen, Vibrational near-field mapping of planar and buried three-dimensional plasmonic nanostructures. Nat. Commun. **4**, 2237 (2013). 10.1038/ncomms3237 23892519 10.1038/ncomms3237 PMC3731659

[13] A. John-Herpin, A. Tittl, L. Kühner, F. Richter, S.H. Huang et al., Metasurface-enhanced infrared spectroscopy: an abundance of materials and functionalities. Adv. Mater. **35**, e2110163 (2023). 10.1002/adma.202110163 35638248 10.1002/adma.202110163

[14] H. Altug, S.H. Oh, S.A. Maier, J. Homola, Advances and applications of nanophotonic biosensors. Nat. Nanotechnol. **17**(1), 5–16 (2022). 10.1038/s41565-021-01045-5 35046571 10.1038/s41565-021-01045-5

[15] X. Hui, C. Yang, D. Li, X. He, H. Huang et al., Infrared plasmonic biosensor with tetrahedral DNA nanostructure as carriers for label-free and ultrasensitive detection of miR-155. Adv. Sci. **8**, e2100583 (2021). 10.1002/advs.202100583 10.1002/advs.202100583 PMC8373097 34155822

[16] A. John-Herpin, D. Kavungal, L. von Mücke, H. Altug, Infrared metasurface augmented by deep learning for monitoring dynamics between all major classes of biomolecules. Adv. Mater. **33**, e2006054 (2021). 10.1002/adma.202006054 33615570 10.1002/adma.202006054 PMC11469153

[17] D. Rodrigo, A. Tittl, N. Ait-Bouziad, A. John-Herpin, O. Limaj et al., Resolving molecule-specific information in dynamic lipid membrane processes with multi-resonant infrared metasurfaces. Nat. Commun. **9**, 2160 (2018). 10.1038/s41467-018-04594-x 29867181 10.1038/s41467-018-04594-x PMC5986821

[18] R. Adato, H. Altug, In-situ ultra-sensitive infrared absorption spectroscopy of biomolecule interactions in real time with plasmonic nanoantennas. Nat. Commun. **4**, 2154 (2013). 10.1038/ncomms3154 23877168 10.1038/ncomms3154 PMC3759039

[19] L. Paggi, A. Fabas, H. El Ouazzani, J.P. Hugonin, N. Fayard et al., Over-coupled resonator for broadband surface enhanced infrared absorption (*SEIRA*). Nat. Commun. **14**, 4814 (2023). 10.1038/s41467-023-40511-7 37558692 10.1038/s41467-023-40511-7 PMC10412556

[20] A. Tittl, A. Leitis, M. Liu, F. Yesilkoy, D.-Y. Choi et al., Imaging-based molecular barcoding with pixelated dielectric metasurfaces. Science **360**, 1105–1109 (2018). 10.1126/science.aas9768 29880685 10.1126/science.aas9768

[21] F. Yesilkoy, E.R. Arvelo, Y. Jahani, M. Liu, A. Tittl et al., Ultrasensitive hyperspectral imaging and biodetection enabled by dielectric metasurfaces. Nat. Photonics **13**, 390–396 (2019). 10.1038/s41566-019-0394-6

[22] A. Aigner, A. Tittl, J. Wang, T. Weber, Y. Kivshar et al., Plasmonic bound states in the continuum to tailor light–matter coupling. Sci. Adv. **8**, eadd4816 (2022). 10.1126/sciadv.add4816 36490330 10.1126/sciadv.add4816 PMC9733921

[23] S. Rosas, K.A. Schoeller, E. Chang, H. Mei, M.A. Kats et al., Metasurface-enhanced mid-infrared spectrochemical imaging of tissues. Adv. Mater. **35**, 2301208 (2023). 10.1002/adma.202301208 10.1002/adma.202301208 PMC10524888 37186328

[24] H. Zhou, D. Li, Z. Ren, C. Xu, L.-F. Wang et al., Surface plasmons-phonons for mid-infrared hyperspectral imaging. Sci. Adv. **10**, eado3179 (2024). 10.1126/sciadv.ado3179 38809968 10.1126/sciadv.ado3179 PMC11135386

[25] D. Rodrigo, O. Limaj, D. Janner, D. Etezadi, F.J. García de Abajo et al., Mid-infrared plasmonic biosensing with graphene. Science **349**, 165–168 (2015). 10.1126/science.aab2051 26160941 10.1126/science.aab2051

[26] C. Wu, A.B. Khanikaev, R. Adato, N. Arju, A. Ali Yanik et al., Fano-resonant asymmetric metamaterials for ultrasensitive spectroscopy and identification ofmolecular monolayers. Nat. Mater. **11**, 69–75 (2012). 10.1038/nmat3161 10.1038/nmat3161 22081082

[27] H. Zhou, X. Hui, D. Li, D. Hu, X. Chen et al., Metal-organic framework-surface-enhanced infrared absorption platform enables simultaneous on-chip sensing of greenhouse gases. Adv. Sci. **7**, 2001173 (2020). 10.1002/advs.202001173 10.1002/advs.202001173 PMC7578855 33101855

[28] X. Miao, T.S. Luk, P.Q. Liu, Liquid-metal-based nanophotonic structures for high-performance *SEIRA* sensing. Adv. Mater. **34**, e2107950 (2022). 10.1002/adma.202107950 34991178 10.1002/adma.202107950

[29] I. Hwang, M. Kim, J. Yu, J. Lee, J.H. Choi et al., Ultrasensitive molecule detection based on infrared metamaterial absorber with vertical nanogap. Small Methods **5**, e2100277 (2021). 10.1002/smtd.202100277 34927875 10.1002/smtd.202100277

[30] J. Yi, E.-M. You, S.-Y. Ding, Z.-Q. Tian, Unveiling the molecule-plasmon interactions in surface-enhanced infrared absorption spectroscopy. Natl. Sci. Rev. **7**, 1228–1238 (2020). 10.1093/nsr/nwaa054 34692147 10.1093/nsr/nwaa054 PMC8288858

[31] Z. Ren, Z. Zhang, J. Wei, B. Dong, C. Lee, Wavelength-multiplexed hook nanoantennas for machine learning enabled mid-infrared spectroscopy. Nat. Commun. **13**, 3859 (2022). 10.1038/s41467-022-31520-z 35790752 10.1038/s41467-022-31520-z PMC9256719

[32] L. Dong, X. Yang, C. Zhang, B. Cerjan, L. Zhou et al., Nanogapped Au antennas for ultrasensitive surface-enhanced infrared absorption spectroscopy. Nano Lett. **17**, 5768–5774 (2017). 10.1021/acs.nanolett.7b02736 28787169 10.1021/acs.nanolett.7b02736

[33] D. Yoo, F. de León-Pérez, M. Pelton, I.-H. Lee, D.A. Mohr et al., Ultrastrong plasmon–phonon coupling via epsilon-near-zero nanocavities. Nat. Photonics **15**, 125–130 (2021). 10.1038/s41566-020-00731-5

[34] D. Li, A. Yadav, H. Zhou, K. Roy, P. Thanasekaran et al., Advances and applications of metal-organic frameworks (MOFs) in emerging technologies: a comprehensive review. Glob. Chall. **8**, 2300244 (2023). 10.1002/gch2.202300244 38356684 10.1002/gch2.202300244 PMC10862192

[35] D. Li, H. Zhou, X. Hui, X. He, H. Huang et al., Multifunctional chemical sensing platform based on dual-resonant infrared plasmonic perfect absorber for on-chip detection of poly(ethyl cyanoacrylate). Adv. Sci. **8**, e2101879 (2021). 10.1002/advs.202101879 10.1002/advs.202101879 PMC8529490 34423591

[36] K. Chen, R. Adato, H. Altug, Dual-band perfect absorber for multispectral plasmon-enhanced infrared spectroscopy. ACS Nano **6**, 7998–8006 (2012). 10.1021/nn3026468 22920565 10.1021/nn3026468

[37] R.A. Maniyara, D. Rodrigo, R. Yu, J. Canet-Ferrer, D.S. Ghosh et al., Tunable plasmons in ultrathin metal films. Nat. Photonics **13**, 328–333 (2019). 10.1038/s41566-019-0366-x

[38] P. Jangid, F.U. Richter, M.L. Tseng, I. Sinev, S. Kruk et al., Spectral tuning of high-harmonic generation with resonance-gradient metasurfaces. Adv. Mater. **36**, e2307494 (2024). 10.1002/adma.202307494 37933748 10.1002/adma.202307494

[39] F.U. Richter, I. Sinev, S. Zhou, A. Leitis, S.H. Oh et al., Gradient high-Q dielectric metasurfaces for broadband sensing and control of vibrational light–matter coupling. Adv. Mater. **36**, e2314279 (2024). 10.1002/adma.202314279 38511549 10.1002/adma.202314279

[40] A. Leitis, A. Tittl, M. Liu, B.H. Lee, M.B. Gu et al., Angle-multiplexed all-dielectric metasurfaces for broadband molecular fingerprint retrieval. Sci. Adv. **5**, eaaw2871 (2019). 10.1126/sciadv.aaw2871 31123705 10.1126/sciadv.aaw2871 PMC6527437

[41] J. Wang, T. Weber, A. Aigner, S.A. Maier, A. Tittl, Mirror-coupled plasmonic bound states in the continuum for tunable perfect absorption. Laser Photonics Rev. **17**, 2300294 (2023). 10.1002/lpor.202300294

[42] Z. Chen, D. Li, H. Zhou, T. Liu, X. Mu, A hybrid graphene metamaterial absorber for enhanced modulation and molecular fingerprint retrieval. Nanoscale **15**, 14100–14108 (2023). 10.1039/d3nr02830e 37581407 10.1039/d3nr02830e

[43] J. Vogt, C. Huck, F. Neubrech, A. Toma, D. Gerbert et al., Impact of the plasmonic near- and far-field resonance-energy shift on the enhancement of infrared vibrational signals. Phys. Chem. Chem. Phys. **17**, 21169–21175 (2015). 10.1039/c4cp04851b 25516198 10.1039/c4cp04851b

[44] J.-H. Park, A. Ndao, W. Cai, L. Hsu, A. Kodigala et al., Symmetry-breaking-induced plasmonic exceptional points and nanoscale sensing. Nat. Phys. **16**, 462–468 (2020). 10.1038/s41567-020-0796-x

[45] R. Adato, A. Artar, S. Erramilli, H. Altug, Engineered absorption enhancement and induced transparency in coupled molecular and plasmonic resonator systems. Nano Lett. **13**, 2584–2591 (2013). 10.1021/nl400689q 23647070 10.1021/nl400689q

[46] H. Zhou, Z. Ren, C. Xu, L. Xu, C. Lee, MOF/polymer-integrated multi-hotspot mid-infrared nanoantennas for sensitive detection of CO_2_ gas. Nano-Micro Lett. **14**, 207 (2022). 10.1007/s40820-022-00950-1 10.1007/s40820-022-00950-1 PMC9588146 36271989

[47] J. Wei, Y. Li, Y. Chang, D.M.N. Hasan, B. Dong et al., Ultrasensitive transmissive infrared spectroscopy via loss engineering of metallic nanoantennas for compact devices. ACS Appl. Mater. Interfaces **11**, 47270–47278 (2019). 10.1021/acsami.9b18002 31769956 10.1021/acsami.9b18002

[48] D. Li, H. Zhou, Z. Chen, Z. Ren, C. Xu et al., Ultrasensitive molecular fingerprint retrieval using strongly detuned overcoupled plasmonic nanoantennas. Adv. Mater. **35**, e2301787 (2023). 10.1002/adma.202301787 37204145 10.1002/adma.202301787

[49] H. Zhou, D. Li, Z. Ren, X. Mu, C. Lee, Loss-induced phase transition in mid-infrared plasmonic metamaterials for ultrasensitive vibrational spectroscopy. InfoMat **4**, e12349 (2022). 10.1002/inf2.12349

[50] J. Xu, Z. Ren, B. Dong, X. Liu, C. Wang et al., Nanometer-scale heterogeneous interfacial sapphire wafer bonding for enabling plasmonic-enhanced nanofluidic mid-infrared spectroscopy. ACS Nano **14**, 12159–12172 (2020). 10.1021/acsnano.0c05794 32812748 10.1021/acsnano.0c05794

[51] H. Zhou, Z. Ren, D. Li, C. Xu, X. Mu et al., Dynamic construction of refractive index-dependent vibrations using surface plasmon-phonon polaritons. Nat. Commun. **14**, 7316 (2023). 10.1038/s41467-023-43127-z 37952033 10.1038/s41467-023-43127-z PMC10640644

[52] B. Cerjan, X. Yang, P. Nordlander, N.J. Halas, Asymmetric aluminum antennas for self-calibrating surface-enhanced infrared absorption spectroscopy. ACS Photonics **3**, 354–360 (2016). 10.1021/acsphotonics.6b00024

[53] K. Chen, T.D. Dao, S. Ishii, M. Aono, T. Nagao, Infrared aluminum metamaterial perfect absorbers for plasmon-enhanced infrared spectroscopy. Adv. Funct. Mater. **25**, 6637–6643 (2015). 10.1002/adfm.201501151

[54] F. Neubrech, A. Pucci, T.W. Cornelius, S. Karim, A. García-Etxarri et al., Resonant plasmonic and vibrational coupling in a tailored nanoantenna for infrared detection. Phys. Rev. Lett. **101**, 157403 (2008). 10.1103/PhysRevLett.101.157403 18999639 10.1103/PhysRevLett.101.157403

[55] M. Bertolotti, Waves and fields in optoelectronics. Opt. Acta Int. J. Opt. **32**, 748 (1985). 10.1080/716099690

[56] P.A. Thomas, W.J. Tan, H.A. Fernandez, W.L. Barnes, A new signature for strong light–matter coupling using spectroscopic ellipsometry. Nano Lett. **20**, 6412–6419 (2020). 10.1021/acs.nanolett.0c01963 32709208 10.1021/acs.nanolett.0c01963 PMC7608940

[57] M.F. Limonov, M.V. Rybin, A.N. Poddubny, Y.S. Kivshar, Fano resonances in photonics. Nat. Photonics **11**(9), 543–554 (2017). 10.1038/nphoton.2017.142

[58] I. Dolado, C. Maciel-Escudero, E. Nikulina, E. Modin, F. Calavalle et al., Remote near-field spectroscopy of vibrational strong coupling between organic molecules and phononic nanoresonators. Nat. Commun. **13**, 6850 (2022). 10.1038/s41467-022-34393-4 36369225 10.1038/s41467-022-34393-4 PMC9652397

[59] H. Durmaz, Y. Li, A.E. Cetin, A multiple-band perfect absorber for *SEIRA* applications. Sens. Actuat. B Chem. **275**, 174–179 (2018). 10.1016/j.snb.2018.08.053

[60] L. Liu, S. Iketani, Y. Guo, J.F.-W. Chan, M. Wang et al., Striking antibody evasion manifested by the *Omicron* variant of SARS-CoV-2. Nature **602**, 676–681 (2022). 10.1038/s41586-021-04388-0 35016198 10.1038/s41586-021-04388-0

[61] F. Zhu, C. Zhuang, K. Chu, L. Zhang, H. Zhao et al., Safety and immunogenicity of a live-attenuated influenza virus vector-based intranasal SARS-CoV-2 vaccine in adults: randomised, double-blind, placebo-controlled, phase 1 and 2 trials. Lancet Respir. Med. **10**, 749–760 (2022). 10.1016/S2213-2600(22)00131-X 35644168 10.1016/S2213-2600(22)00131-X PMC9135375

[62] J. Cheong, H. Yu, C.Y. Lee, J.U. Lee, H.J. Choi et al., Fast detection of SARS-CoV-2 RNA via the integration of plasmonic thermocycling and fluorescence detection in a portable device. Nat. Biomed. Eng. **4**, 1159–1167 (2020). 10.1038/s41551-020-00654-0 33273713 10.1038/s41551-020-00654-0 PMC8202505

[63] L. Wang, X. Wang, Y. Wu, M. Guo, C. Gu et al., Rapid and ultrasensitive electromechanical detection of ions, biomolecules and SARS-CoV-2 RNA in unamplified samples. Nat. Biomed. Eng. **6**, 276–285 (2022). 10.1038/s41551-021-00833-7 35132229 10.1038/s41551-021-00833-7

[64] D. Li, H. Zhou, X. Hui, X. He, X. Mu, Plasmonic biosensor augmented by a genetic algorithm for ultra-rapid, label-free, and multi-functional detection of COVID-19. Anal. Chem. **93**, 9437–9444 (2021). 10.1021/acs.analchem.1c01078 34170680 10.1021/acs.analchem.1c01078 PMC8262173

[65] S.X. Leong, Y.X. Leong, E.X. Tan, H.Y.F. Sim, C.S.L. Koh et al., Noninvasive and point-of-care surface-enhanced Raman scattering (SERS)-based Breathalyzer for mass screening of coronavirus disease 2019 (COVID-19) under 5 min. ACS Nano **16**, 2629–2639 (2022). 10.1021/acsnano.1c09371 35040314 10.1021/acsnano.1c09371

[66] H. Yao, Y. Song, Y. Chen, N. Wu, J. Xu et al., Molecular architecture of the SARS-CoV-2 virus. Cell **183**, 730-738.e13 (2020). 10.1016/j.cell.2020.09.018 32979942 10.1016/j.cell.2020.09.018 PMC7474903

[67] X.X. Han, R.S. Rodriguez, C.L. Haynes, Y. Ozaki, B. Zhao, Surface-enhanced Raman spectroscopy. Nat. Rev. Meth. Primers **1**, 87 (2022). 10.1038/s43586-021-00083-6

[68] X. Liu, Z. Zhang, J. Zhou, W. Liu, G. Zhou et al., Artificial intelligence-enhanced waveguide “photonic nose”-augmented sensing platform for VOC gases in mid-infrared. Small **20**, e2400035 (2024). 10.1002/smll.202400035 38576121 10.1002/smll.202400035

[69] W. Liu, Y. Ma, X. Liu, J. Zhou, C. Xu et al., Larger-than-unity external optical field confinement enabled by metamaterial-assisted comb waveguide for ultrasensitive long-wave infrared gas spectroscopy. Nano Lett. **22**, 6112–6120 (2022). 10.1021/acs.nanolett.2c01198 35759415 10.1021/acs.nanolett.2c01198

[70] J. Zhou, Z. Zhang, B. Dong, Z. Ren, W. Liu et al., Midinfrared spectroscopic analysis of aqueous mixtures using artificial-intelligence-enhanced metamaterial waveguide sensing platform. ACS Nano **17**, 711–724 (2023). 10.1021/acsnano.2c10163 36576121 10.1021/acsnano.2c10163

[71] J. Hu, D. Mengu, D.C. Tzarouchis, B. Edwards, N. Engheta et al., Diffractive optical computing in free space. Nat. Commun. **15**, 1525 (2024). 10.1038/s41467-024-45982-w 38378715 10.1038/s41467-024-45982-w PMC10879514

[72] Z. Zhang, X. Liu, H. Zhou, S. Xu, C. Lee, Advances in machine-learning enhanced nanosensors: from cloud artificial intelligence toward future edge computing at chip level. Small Struct. **5**, 2300325 (2024). 10.1002/sstr.202300325

[73] A. Bylinkin, M. Schnell, M. Autore, F. Calavalle, P. Li et al., Real-space observation of vibrational strong coupling between propagating phonon polaritons and organic molecules. Nat. Photonics **15**, 197–202 (2021). 10.1038/s41566-020-00725-3

