# Supplementary material for: Tailoring Light–Matter Interactions in Overcoupled Resonator for Biomolecule Recognition and Detection
Source: Nanomicro Lett. 2024 Sep 26;17:10. doi: 10.1007/s40820-024-01520-3 (PMC11427657; doi:10.1007/s40820-024-01520-3)
Supplement: Supplementary file 1 — Supplementary file1 (DOCX 25350 kb) [file 40820_2024_1520_MOESM1_ESM.docx]

Supporting Information for

**Tailoring Light-Matter Interactions in Overcoupled Resonators for Biomolecule Recognition and Detection**

Dongxiao Li^1,2^, Hong Zhou^1,2^, Zhihao Ren^1,2^, Cheng Xu^1,2^, Chengkuo Lee^1,2,^*

^1^ Department of Electrical and Computer Engineering, National University of Singapore, 117583, Singapore

^2^ Center for Intelligent Sensors and MEMS, National University of Singapore, 117608, Singapore

*Corresponding author. E-mail: [elelc@nus.edu.sg](mailto:elelc@nus.edu.sg) (Chengkuo Lee)

**S1 Numerical simulations**

Finite-difference time-domain (FDTD Solutions ver. 8.19, Lumerical) software was used for numerical simulations. The light source was a vertically incident plane wave, and the polarization state was along the long axis of the nanorod antenna. To improve simulation efficiency, periodic boundary conditions were used in the *x* and *y* directions, and perfect matching layer (PML) boundary conditions were used in the *z*-direction. The complex refractive index of Al is taken from CRC *et al*. [S1], and the complex refractive index of Al_2_O_3_ is taken from Palik *et al*. [S2] The complex refractive index of PMMA is taken from Zhang *et al*. [S3] The complex refractive index of proein is taken from Durmaz *et al*. [S4] Use a 3D frequency-domain power monitor to simulate near-field distribution. The 5 nm thick Ti adhesion layer was omitted in the simulation.

**S2** **Temporal coupled model theory**

Temporal coupled-mode theory (TCMT) has been widely used to describe light-matter interactions in resonator-molecule coupled systems [S5]. Here, we understand and maximize the surface-enhanced infrared absorption (SEIRA) performance of plasmonic resonators based on TCMT. Metamaterial absorber (MA) with a three-layer structure was chosen for discussion because it is easier to obtain overcoupled (OC) resonator. In addition, MA have greater light-plasmon coupling strength compared with single-layer nanoantennas, which will achieve greater SEIRA sensitivity [S6]. In the MA-molecule coupling system, the general expression of TCMT is as follows:

$\frac{d}{dt}P=j\omega_{0}P-\left(\gamma_{r}+\gamma_{a}\right)P+\kappa S_{1+}+j\mu M$ (S1)

$\frac{d}{dt}M=j\omega_{m}M-\gamma_{m}M+j\mu P$ (S2)

$S_{1-}=-S_{1+}+\kappa P$ (S3)

$A\text{=}1-R-T\text{=1}-\left|r\right|^{2}=1-\left|\frac{S_{1-}}{S_{1+}}\right|^{2}$ (S4)

where *P* is the mode amplitudes of MA, and *M* is the mode amplitudes of the molecular vibration. *ω_0_* is the resonance frequency of MA, and *ω_m_* is the resonance frequency of the molecular vibration. *γ_r_* and *γ_a_* represent the radiative and absorptive losses of MA, respectively. *γ_m_* is the absorptive loss of molecules. *κ* is the coupling coefficient between light and MA. In the MA, *κ*=(2*γ_r_*)^1/2^. *μ* is the coupling coefficient between MA and molecules. *S_1+_* and *S_1-_* are the input and output traveling waves, respectively. *A*, *R*, and *T* represent the absorption, reflection, and transmission spectra of MA respectively. In MA, *T*=0 due to the presence of metal backplate. *r* is the reflection coefficient, which represents the ratio of the output traveling wave to the input traveling wave (*r*=*S_1-_*/*S_1+_*). By derivation, the absorption spectrum (*A*) of MA coupling molecules can be obtained,

$A\text{=1}-\left|\frac{2\gamma_{r}}{j(\omega-\omega_{0})+(\gamma_{r}+\gamma_{a})+\left(\frac{\mu^{2}}{j\left(\omega-\omega_{m}\right)+\gamma_{m}}\right)}-1\right|^{2}$ (S5)

Here, the absorption spectrum of bare MA is obtained when *μ*=0.

$\left.A\right|_{\mu=0}\text{=1}-\left|\frac{2\gamma_{r}}{j(\omega-\omega_{0})+(\gamma_{r}+\gamma_{a})}-1\right|^{2}$ (S6)

The molecular signal enhancement spectrum can be expressed as the difference between the absorption spectrum with and without molecules.

$\Delta A\text{=}\left.A\right|_{\mu\neq0}-\left.A\right|_{\mu=0}$ (S7)

To clarify the signal enhancement situation at the molecular vibration frequency (*ω_m_*), we define the sensitivity of SEIRA as

$\begin{matrix} \left.I_{SEIRA}=\Delta A\right|_{\omega=\omega_{m}}=\left.\left(\left.A\right|_{\mu\neq0}-\left.A\right|_{\mu=0}\right)\right|_{\omega=\omega_{m}} \\ =\left|\frac{2\gamma_{r}}{j(\omega_{m}-\omega_{0})+(\gamma_{r}+\gamma_{a})}-1\right|^{2}-\left|\frac{2\gamma_{r}}{j(\omega_{m}-\omega_{0})+(\gamma_{r}+\gamma_{a})+\left(\frac{\mu^{2}}{\gamma_{m}}\right)}-1\right|^{2} \end{matrix}$ (S8)

Fig. S1 is used to visually demonstrate the definition of *I_SEIRA_*. According to Equation (S8), it can be found that the *I_SEIRA_* is related to six parameters, namely *ω_m_*, *ω_0_*, *γ_r_*, *γ_a_*, *μ*, and *γ_m_*. To simplify the parameter dimensions, we define the spectral detuning as *Δω*=*ω_0_*-*ω_m_*, the ratio of the loss rate as *f=γ_r_*/*γ_a_*, the molecular coupling parameter as *ξ*=*μ*/*γ_m_*. The physical significance of Equation (S8) includes the following three points: 1. Understanding the effect of spectral detuning (*Δω*) on absorption enhancement. 2. Understanding the effect of MA loss parameters (*f*) on absorption enhancement. 3. Understanding the effect of molecular coupling parameters (*ξ*) on absorption enhancement. Here, we fixed *ω_m_*=1750 cm^-1^, *γ_a_*=10 cm^-1^, and *γ_m_*=8 cm^-1^. After dimensionality reduction, we can obtain *I_SEIRA_* as a mapping of *Δω*, *f* and ξ (Fig. 2a, Fig. S2).

Next, we discussed different cases of sensitivity. First, we discuss the case where the sensitivity is 0. To simplify the discussion, we set *ω*=*ω_m_*=*ω*_0_. According to Equation (S8), we plot the sensitivity as a function of *γ_r_*/*γ_a_*, as shown in Fig. S3a. It can be found that when *γ_r_*/*γ_a_* is slightly greater than 1, *I_SEIRA_*=0. In this case, the intensity of the absorption spectrum at *ω*=*ω_m_* does not change significantly when MA couples the molecule (Fig. S3biii). Fig. S3ciii is the corresponding absorption difference spectrum. To clarify the value of *γ_r_*/*γ_a_* when the sensitivity is 0, we derive Equation (S8). The derivation result is as follows,

$\begin{matrix} \left.I_{SEIRA}\right|_{\omega=\omega_{m}=\omega_{0}}=\left.\Delta A\right|_{\omega=\omega_{m}=\omega_{0}}\text{=}\left.\left(\left.A\right|_{\mu\neq0}-\left.A\right|_{\mu=0}\right)\right|_{\omega=\omega_{m}=\omega_{0}} \\ =\frac{4\left(\gamma_{r}\frac{\mu^{2}}{\gamma_{m}(\gamma_{r}+\gamma_{a})^{2}}\right)}{\left((\gamma_{r}+\gamma_{a})+\left(\frac{\mu^{2}}{\gamma_{m}}\right)\right)^{2}}\left(\gamma_{r}^{2}-\gamma_{a}^{2}-\frac{\mu^{2}\gamma_{a}}{\gamma_{m}}\right) \end{matrix}$ (S9)

When the sensitivity is 0, that is,

$\gamma_{r}^{2}-\gamma_{a}^{2}-\frac{\mu^{2}\gamma_{a}}{\gamma_{m}}=0$ (S10)

By derivation, we can get

$\frac{\gamma_{r}}{\gamma_{a}}=\sqrt{1+\frac{\mu^{2}}{\gamma_{m}\gamma_{a}}}$ (S11)

Therefore, when *γ_r_*/*γ_a_* satisfies Equation (S11), the sensitivity is 0.

Next, we discussed the cases where *I_SEIRA_*>0 and *I_SEIRA_*<0. In Fig. S3a, we found that there are two regions of sensitivity. Among them, the sensitivity of the blue area on the left is less than 0, and the sensitivity of the red area on the right is greater than 0. This phenomenon is related to two effects, namely electromagnetically induced transparency (EIT) and electromagnetically induced absorption (EIA) [S7]. For intuitive display, we select two points in the blue area and the red area respectively, and show their absorption spectra (Fig. S3b) and absorption difference spectra (Fig. S3c). In the blue area, the absorption spectrum after MA coupling molecules shows a downward dip (Fig. S3bi, S3bii). This is a typical EIT effect. Affected by this effect, the absorption difference spectrum is negative when *ω*=*ω_m_* (Fig. S3ci, S3cii). On the contrary, the absorption spectrum after MA coupling molecules shows an upward dip in the red area (Fig. S3biv, S3bv). This phenomenon is also called the EIA effect. Under the influence of EIA effect, the absorption difference spectrum is positive when *ω*=*ω_m_* (Fig. S3civ, S3cv).

**S3 Device Fabrication**

The 4-inch wafers were sonicated with acetone for 3 mins and then rinsed in isopropanol (IPA) followed by nitrogen drying. After drying, 5 nm thick Ti and 100 nm thick Al were sequentially deposited on the silicon wafer through an e-beam evaporator (ATC-T Series, AJA Int.). Among them, Ti was used as an adhesion layer to enhance the adhesion between the Al film and the Si substrate. Divide the silicon wafer prepared above into two and use an e-beam evaporator to deposit 100 nm and 550 nm thick Al_2_O_3_ on them respectively. Among them, 100 nm thick Al_2_O_3_ was used to prepare UC-Hμ devices, and 550 nm thick Al_2_O_3_ was used to prepare OC and OC-Hμ devices. Next, positron e-beam resist PMMA (495k PMMA A4) was coated on the substrate with speed of 4000 rpm for 1 min. Then, the devices were baked on a hotplate at 180℃ for 3 mins. After heating, a commercial electron-conducting polymer the thin conductive polymer (Espacer 300Z from Showa Denko Singapore) was spin-coated at 2000 rpm for 40 s to eliminate charge accumulation during e-beam exposure. Patterned nanoantennas were prepared using e-beam lithography (Raith GmbH). After exposure, the devices were immersed in deionized water for 15 s to remove Espacer, then developed in a MIBK/IPA (1:3) mixture for 50 s, and finally rinsed in IPA for 30 s. Then, 5 nm thick Ti and 60 nm Al were deposited on the device using an e-beam evaporator. Finally, the devices were soaked in acetone for 24 hours to remove unexposed PMMA to obtain the final nanoantenna pattern. These devices were stored in a dry and sealed environment before use.

**Supplementary Figures and Tables**

**Fig. S1** Visual demonstration of sensitivity (*I_SEIRA_*) when *ω_m_*=*ω*_0_ (a) and *ω_m_*≠*ω*_0_ (b). Here, we set *γ_r_*=50 cm^-1^, *γ_a_*=10 cm^-1^, *μ*=8 cm^-1^, *γ_m_*=8 cm^-1^. Among them, the black dashed curve is the absorption spectrum of MA without coupling molecules, the red solid curve is the absorption spectrum of MA after coupling molecules, and the blue arrow is the sensitivity of SEIRA

**Fig. S2** Calculated 2D mapping of *I_SEIRA_* as a function of *Δω* and *γ_r_*/*γ_a_*. (I)-(VIII) respectively represent different *μ*, and the range of *μ* is from 2 cm^-1^ to 24 cm^-1^. Here, we fixed the absorption loss of the molecule *γ_m_*=8 cm^-1^. All mappings use the same color scale

**Fig. S3** **a** MA sensitivity as a function of *γ_r_*/*γ_a_* when spectrally matched (*ω_m_*=*ω*_0_). We set *ω_m_*=*ω*_0_=1750 cm^-1^, *γ_a_*=10 cm^-1^, *μ*=8 cm^-1^, *γ_m_*=8 cm^-1^. **b** Calculated the absorption spectra of MA before (dashed curves) and after (solid curves) coupling with molecules. The corresponding *γ_r_*/*γ_a_* for (i)-(v) are 0.07, 0.35, 1.34, 5, and 23, respectively. **c** Extracted the absorption difference spectra from (b)

**Fig. S4** Investigate the relationship between the broadband of OC mode and *γ_r_*/*γ_a_*. **a** Full width at half maximum (FWHM) of MA as function of *γ_r_*/*γ_a_*. Both *x*- and *y*-axis use logarithmic scale. **b** The absorption spectra of five MAs were calculated, and the ratios of their loss rates were *γ_r_*/*γ_a_*=0.1 (black), *γ_r_*/*γ_a_*=0.5 (red), *γ_r_*/*γ_a_*=1 (blue), *γ_r_*/*γ_a_*=5 (green), and *γ_r_*/*γ_a_*=10 (purple), respectively. The FWHM of MA increase with the increase of *γ_r_*/*γ_a_*. In particular, MA has a larger FWHM in the overcoupled (OC, *γ_r_*/*γ_a_*>1) mode compared to the undercoupled (UC, *γ_r_*/*γ_a_*<1) mode. Larger FWHM enables wider enhancement bandwidth for OC mode

**Fig. S5** Study the relationship between SEIRA sensitivity, MA absorption spectrum intensity and Q factor. **a** Calculated maximum absorption (black curve) and Q factor (blue curve) as a function of *γ_r_*/*γ_a_*. **b** Calculated SEIRA sensitivity as a function of *γ_r_*/*γ_a_* when spectral match (*ω_0_*=*ω_m_*). The *x*-axis use a logarithmic scale. Comparing (a) and (b), it can be found that the sensitivity of SEIRA is independent of the maximum absorption intensity and Q factor of MA. It should be noted that we set *ω_m_*=*ω*_0_=1750 cm^-1^, *γ_a_*=10 cm^-1^, *μ*=24 cm^-1^, *γ_m_*=8 cm^-1^, and the range of *γ_r_*/*γ_a_* is 0.01~100

**Fig. S6** **a-h** The real and imaginary parts as a function of wavenumber in Fig. 2e. γ_r_/γ_a_=10, corresponding to the OC mode. *µ/γ_m_* ranges from 0 to 3.5, where *γ_m_*=8 cm^-1^. Wavenumber range from 750 cm^-1^ to 2750 cm^-1^

**Fig. S7** **a** Complex representation of Fresnel reflection in UC (γ*_r_*/γ*_a_*=0.4) mode. A secondary loop appears in Fig. S7 when *µ/γ_m_*>1. The generation of secondary loop indicates that the coupled system changes from weak coupling to strong coupling state. **b-i** Real and imaginary parts of the reflection coefficient as a function of wavenumber at different *µ/γ_m_*. *µ/γ_m_* ranges from 0 to 3.5, where *γ_m_*=8 cm^-1^. Wavenumber range from 750 cm^-1^ to 2750 cm^-1^

**Fig. S8** **a** Schematic diagram of the OC device. **b** Simulated damping rate (*γ_r_* (red), *γ_a_* (blue)) as a function of Al_2_O_3_ thickness. Dimensions: *P_x_*=*P_y_*=3.5 μm, *L*=2 μm, *w*=0.7 μm, *t_1_*=*t_2_*=0.1 μm, *h* range from 0.05 μm to 1 μm. As the thickness of the dielectric layer increases, the radiative loss is gradually greater than the absorption loss. **c** Simulated damping rate (*γ_r_* (red), *γ_a_* (blue)) as a function of period, where *P_x_*=*P_y_*. Dimensions: *L*=0.9×*P_x_*, *w*=0.3 μm, *t_1_*=*t_2_*=0.1 μm, *h*=0.5 μm. As the period decreases, *γ_r_*/*γ_a_* further increases. **d** Simulated absorption spectra as a function of antenna gap. Dimensions: *P_x_*=*P_y_*=1 μm, *w*=0.3 μm, *t_1_*=*t_2_*=0.1 μm, *h*=0.5 μm, *L* range from 0.91 μm to 0.99 μm, gap= *P_x_*−*L*. **e** Simulated absorption spectra as a function of antenna width. Dimensions: *P_x_*=*P_y_*=1 μm, *L*=0.95 μm, *t_1_*=*t_2_*=0.1 μm, *h*=0.5 μm, *w* range from 0.1 μm to 0.9 μm. **f** Simulated absorption spectra as a function of *P_y_*. Dimensions: *P_x_*=1 μm, *L*=0.95 μm, *w*=0.3 μm, *t_1_*=*t_2_*=0.1 μm, *h*=0.5 μm, *P_y_* range from 0.8 μm to 2 μm. TCMT was used to extract loss parameters (*γ_r_*/*γ_a_*) of OC-Hµ from (d)-(f). Considering the fabrication process, we used the method of changing the *P_y_* dimension to modulate the resonance frequency of OC-Hµ

**Fig. S9** Investigate the effect of the number of molecules on the coupling coefficient *μ*. **a** Schematic diagram of the unit cell of the OC device. Dimensions: *P_x_*=*P_y_*=2.5 μm, *L*=1.5 μm, *w*=0.9 μm, *t_1_*=0.1 μm, *t_2_*=0.05 μm, *h*=0.55 μm. **b** Schematic diagram of increasing molecular layer thickness. The thickness of the PMMA molecular layer ranges from 5 nm to 90 nm. The number of molecules is usually related to film thickness or species concentration. Here, we explore the effect of the number of molecules on the coupling coefficient *μ* by changing the film thickness. The film here uses polymethyl methacrylate (PMMA) molecules. **c** Complex permittivity of C=O band of PMMA. **d** Simulated absorption spectra (solid curves) of OC devices loaded with PMMA molecular layers of different thicknesses. The corresponding dashed curves are the theoretical fitting results. The basis of theoretical fitting is Equation (S5). **e** Extracted *γ_r_* (black), *γ_a_* (red) and *γ_r_*/*γ_a_* (blue) as a function of molecular layer thickness from (d). **f** Extracted *μ* (red), *γ_m_* (black) and *μ*/*γ_m_* (blue) as a function of molecular layer thickness from (d). As the sample thickness increases, there is a competitive relationship between the coupling coefficient μ and the molecular loss rate γ_m_, which can be explained by energy dissipation. In the plasmonic-molecular coupled system, energy is dissipated through plasmonic absorption and scattering, as well as through transfer to and subsequent dissipation by the molecules. The energy dissipation occurs in two main ways: during the transfer process, related to μ, and through molecular absorption, related to γ_m_. With increased sample thickness, more energy is dissipated during transfer, reducing the energy dissipated via molecular absorption. **g** Extracted SEIRA signal strength as a function of *μ*/*γ_m_*. SEIRA signal strength increases with increasing *μ*/*γ_m_*. **h** The complex representation of Fresnel reflection. As the thickness of the molecular layer increases, secondary loops appear in the Fresnel reflection. The emergence of secondary loops indicates that the MA-molecule coupling system enters a strong coupling state

**Fig. S10** Investigate the effect of the gap between adjacent nanoantennas on the coupling coefficient *μ*. **a** Schematic diagram of the unit cell used to demonstrate the effect of the gap on the coupling coefficient *μ*. The gap range is from 20 nm to 200 nm. Dimensions: *P_x_*=0.9+gap μm, *P_y_*=1.25 μm, *L*=0.9 μm, *w*=0.1+gap μm, *t_1_*=0.1 μm, *t_2_*=0.05 μm, *h*=0.55 μm. **b** Schematic diagram of reducing the gap between adjacent nanoantennas. 8 nm thick PMMA was used as the probe molecule. **c** Simulated near-field strength as a function of antenna gap. As the antenna gap decreases, the near-field intensity increases significantly. **d** Simulated the absorption spectra (solid curves) of OC loaded PMMA under different antenna gaps. The corresponding dashed curves are the theoretical fitting results. The basis of theoretical fitting is Equation (S5). **e** Extracted *γ_r_* (black), *γ_a_* (red) and *γ_r_*/*γ_a_* (blue) as a function of antenna gaps from (d). **f** Extracted *μ* (red), *γ_m_* (black) and *μ*/*γ_m_* (blue) as a function of antenna gaps from (d). As the antenna gap decreases, *μ* gradually increases, and *γ_m_* gradually decreases, resulting in *μ*/*γ_m_* gradually increasing. This trend can also be explained from the perspective of energy dissipation. As the gap decreases, the coupling between the antennas strengthens, leading to increased energy dissipation during the transfer process and a reduction in the energy dissipated through molecular absorption. **g** Extracted SEIRA signal strength as a function of *μ*/*γ_m_*. SEIRA signal strength increases with increasing *μ*/*γ_m_*. **h** The complex representation of Fresnel reflection. As the gap between adjacent nanoantennas increases, secondary loops appear in the Fresnel reflection. The emergence of secondary loops indicates that the MA-molecule coupling system enters a strong coupling state

**Fig.** **S11** Compare the spectral lossless reduction characteristics of OC-Hµ and UC-Hµ devices. **a** Fingerprint absorption spectrum (i), first derivative spectrum (ii) and second derivative spectrum (iii) of molecules. **b** The absorption difference spectra (i), first derivative spectra (iii) and second derivative spectra (iii) of the UC-Hµ (*γ_r_*/*γ_a_*=0.4, *γ_a_*=10 cm^-1^, *μ*/*γ_m_*=2.25, *γ_m_*=8 cm^-1^) device after coupling molecules were calculated as a mapping of spectral detuning. **c** The absorption difference spectra (i), first derivative spectra (iii) and second derivative spectra (iii) of the OC-Hµ (*γ_r_*/*γ_a_*=10, *γ_a_*=10 cm^-1^, *μ*/*γ_m_*=2.25, *γ_m_*=8 cm^-1^) device after coupling molecules were calculated as a mapping of spectral detuning. The spectra of *Δω*=0 cm^-1^, 35 cm^-1^ and 70 cm^-1^ were extracted to show the details. The results show that OC-Hµ can directly obtain fingerprint spectra and derivative spectra consistent with molecular vibrations because there is no interference from asymmetric Fano resonance

**Fig. S12** **a** Measured the thickness of PMMA using atomic force microscopy (AFM). First, we spin-coated 0.2% 495 K PMMA in anisole onto the device surface at 4000 rpm for 1 min. The device was then baked on a hot plate at 180°C for 3 mins. After heating, a commercial conductive polymer (Espacer 300Z from Showa Denko Singapore) was spin-coated at 2000 rpm for 40 s to eliminate charge accumulation during electron beam exposure. Patterning was carried out using electron beam lithography. After patterning, the device was soaked in deionized water for 15 s to remove the Espacer, then developed in a MIBK/IPA (1:3) mixture for 50 s, and finally rinsed in IPA for 30 s. After development, a height difference appeared on the PMMA surface. Next, AFM was used to characterize the thickness of the PMMA. **b** Extracted the height curve from (a)

**Fig. S13** **a-c** Theoretical fitting (red curves) of the absorption spectra of the devices loaded with PMMA based on TCMT. The measured spectra (black curves) are from Fig. 3. To simplify the fitting, we only consider the C=O bond vibration in PMMA. The fitting results are shown in Table S3

**Fig. S14** **a** The complex representation of Fresnel reflection of UC-Hµ2 (blue), OC-2 (black) and OC-Hµ2 (red) devices. **b** The complex representation of Fresnel reflection of UC-Hµ3 (blue), OC-3 (black) and OC-Hµ3 (red) devices

**Fig. S15** Principal component analysis (PCA) processing results in Fig. 4e. **a** Loadings of the first three principal components. **b** Explained variance of PCA

**Fig. S16** **a** Absorption difference spectra of OC-Hμ loaded with different glucose concentrations. **b** Extracted SEIRA intensity at 1076 cm^-1^ as a function of glucose concentration

**Fig. S17** **a**-**d** Signal enhancement of different molecules at different wavelengths. Langmuir EXT1 was used to fit the signal enhancement. The formula of Langmuir EXT1 is *y=abx^1-c^/(1+bx^1-c^)*

**Fig. S18** **a** Schematic diagram of the UC-Hµ device. Dimensions: *P_x_*=*P_y_*, which ranges from 0.85 μm to 2 μm, *L_1_*=*L_2_*=*P_x_*-40 nm, *t_1_*=100 nm, *h*=50 nm, *t_2_*=50 nm. The protein layer is 10 nm thick and covers the antenna surface. **b** Simulated absorption spectra of UC-Hµ as a mapping of period. **c** Simulated the absorption spectra of UC-Hµ after protein loading as a mapping of the period. **d** Schematic diagram of the OC-Hµ device. Dimensions: *P_x_*=0.8 μm, *P_y_* range from 0.45 μm to 2.4 μm, *L_1_*=*P_x_*-40 nm, *w*=0.3 μm, *t_1_*=100 nm, *h*=550 nm, *t_2_*=50 nm. The protein layer is 10 nm thick and covers the antenna surface. **e** Simulated absorption spectra of OC-Hµ as a mapping of period *P_y_*. **f** Simulated the absorption spectra of OC-Hµ after protein loading as a mapping of the period *P_y_*

**Fig. S19** PCA processing results in Fig. 6d. **a** The weight of each spectral fraction in two-dimensional space. Each cluster represents a mixing ratio. **b** Loadings of the first three principal components. **c** Explained variance of PCA

**Fig. S20** Multimodal deep neural network (MM-DNN) processing results in Fig. 6h. **a** Loss of the MM-DNN model. **b** Accuracy of the MM-DNN model

**Fig. S21** Measured the spectral noise 20 times consecutively on a pure Au surface using FTIR. Then, we calculated the range of noise using the maximum and minimum values. The range of noise is σ=0.18%. Next, we used LOD=3σ/S and the sensitivity (S) from Table S3 to calculate the LOD

**Table S1** Structure size of different devices. Unit: μm, (except Q and A_max_)

| Device | *h* | *t_1_* | *t_2_* | *P_x_* | *P_y_* | *L* | *w* | *ω_0_* | FWHM | Q | A_max_ |
| --- | --- | --- | --- | --- | --- | --- | --- | --- | --- | --- | --- |
| UC-Hµ1 | 0.1 | 0.1 | 0.06 | 1.2 | 1.2 | 1.16 | 0.8 | 5.44 | 0.70 | 7.76 | 0.82 |
| UC-Hµ2 | 0.1 | 0.1 | 0.06 | 1.4 | 1.4 | 1.36 | 0.6 | 5.85 | 0.68 | 8.64 | 0.81 |
| UC-Hµ3 | 0.1 | 0.1 | 0.06 | 2.0 | 2.0 | 1.96 | 0.6 | 7.41 | 0.74 | 10.06 | 0.65 |
| OC-1 | 0.55 | 0.1 | 0.06 | 2.5 | 2.5 | 1.4 | 1.1 | 5.77 | 1.66 | 3.48 | 0.25 |
| OC-2 | 0.55 | 0.1 | 0.06 | 2.5 | 2.5 | 1.6 | 1.3 | 6.79 | 1.86 | 3.65 | 0.22 |
| OC-3 | 0.55 | 0.1 | 0.06 | 2.5 | 2.5 | 1.8 | 1.5 | 7.73 | 2.01 | 3.85 | 0.23 |
| OC-Hµ1 | 0.55 | 0.1 | 0.06 | 0.95 | 1.4 | 0.91 | 0.3 | 5.41 | 1.91 | 2.84 | 0.31 |
| OC-Hµ2 | 0.55 | 0.1 | 0.06 | 0.95 | 1.0 | 0.91 | 0.3 | 6.04 | 2.14 | 2.83 | 0.23 |
| OC-Hµ3 | 0.55 | 0.1 | 0.06 | 0.95 | 0.6 | 0.91 | 0.3 | 7.19 | 2.95 | 2.44 | 0.19 |

**Table S2** Comparison of SEIRA signals using PMMA as probe molecule. It should be noted that the limit of detection (LOD) of PMMA is calculated based on LOD=3σ/S. Among them, σ is the spectral noise, and S is the sensitivity. For a fair comparison, we used consistent spectral noise to calculate the LOD of PMMA. The measured spectral noise is shown in Fig. S21

| References | Device Type | Sensitivity | Enhance bandwidth | Spectral signal | Spectral versatility | Spectral anti-distortion | LOD | Size |
| --- | --- | --- | --- | --- | --- | --- | --- | --- |
| Wang et al.[S8] | MA, Bound states in the continuum, UC, CC, OC | 3.3%/nm (UC); 1.13%/nm (CC); 4.7%/nm (OC) | 1200–2000 cm^−1^ (arrays) | - | Yes | Yes | 0.164 nm (UC); 0.478 nm (CC); 0.115 nm (OC) | 60 arrays, each array size is 115×115 µm^2^ |
| Aigner et al.[S9] | Plasmonic bound states in the continuum, UC, CC, OC | 1.2%/nm | Enhance C=O band | - | Yes | - | 0.45 nm | 64 arrays, total size is 2 mm^2^ |
| Tittl et al.[S10] | Pixelated dielectric metasurfaces | - | - | - | Yes | Yes | - | 100 arrays, each array size is 100×100 µm^2^ |
| Yue et al.[S11] | MA, Multiple-resonant pad-rod nanoantennas | 0.29%/nm | Enhance C=O band, C-H band | - | No | No | 1.862 nm | - |
| Paggi et al.[S12] | MA, OC, Helmholtz optical resonator | 3.1%/nm | 3-10 µm | - | Yes | - | 0.174 nm | 100×100 µm^2^ |
| Habeeb et al.[S13] | GZO nano-triangle | 0.06%/nm | - | Asymmetric Fano lineshape | No | No | 9 nm | - |
| Chen et al.[S14] | MA, CC mode, dual-resonant perfect absorber | 2.5%/nm | 5-6.6 μm;  3.2-3.8 μm | Asymmetric Fano lineshape at weak detuning | No | No | 0.216 nm | - |
| Chen et al.[S15] | Asymmetric Babinet-inverted Fano-resonant | 0.7%/nm | Enhance C=O band | Asymmetric Fano lineshape at weak detuning | No | No | 0.771 nm | 50×50 µm^2^ |
| Lahiri et al.[S16] | Asymmetric split ring resonators | 0.15%/nm | Enhance C=O band | Asymmetric Fano lineshape at weak detuning | No | No | 3.6 nm | - |
| Hoffmann et al.[S17] | Bowties | 0.09%/nm | Enhance C=O band | - | No | No | 6 nm | - |
| Petefish et al.[S18] | Grating-Coupled Surface Plasmon Resonance | 0.14%/nm | Enhance C=O band | - | No | No | 3.86 nm | - |
| Wang et al.[S19] | Gold strip gratings | 0.2%/nm | Enhance C=O band | Asymmetric Fano lineshape at weak detuning | No | No | 2.7 nm | 160×85 µm^2^ |
| Durmaz et al.[S4] | MA, CC mode, multiple-band perfect absorber | 1%/nm | 4-7 μm | - | No | - | 0.54 nm | 100×100 µm^2^ |
| Cetin et al.[S20] | MA, CC mode, dual-resonant perfect absorber | 1.8%/nm | 5-6.6 μm;  3.2-3.8 μm | Asymmetric Fano lineshape at weak detuning | No | No | 0.3 nm | - |
| Li et al.[S21] | Graphene nanoribbon | 0.09%/nm | Enhance C=O band | Asymmetric Fano lineshape at weak detuning | No | No | 6 nm | - |
| Abb et al.[S22] | Metal oxide plasmonic antenna | 0.16%/nm | - | Asymmetric Fano lineshape at weak detuning | - | No | 3.38 nm | 120×120 µm^2^ |
| Zhou et al.[S23] | Multi‑hotspot mid‑infrared nanoantennas | 0.46%/nm | Enhance C=O band | - | No | No | 1.17 nm | 100×100 µm^2^ |
| Wei et al.[S24] | Crooked nanoantennas | 1.4%/nm | Enhance C=O band | Asymmetric Fano lineshape at weak detuning | No | No | 0.386 nm | - |
| Ren et al.[S25] | Wavelength-multiplexed hook nanoantenna | 0.8%/nm | 6-9 µm | Asymmetric Fano lineshape at weak detuning | Yes | No | 0.675 nm | 200×200 µm^2^, single array |
| This work | MA, UC-Hµ | 1.89%/nm | 5.2-6.3 µm (*ω_0_*=5.78 µm) | Asymmetric Fano lineshape at weak detuning | No | No | 0.286 nm | 100×100 µm^2^, single array |
|  | MA, OC | 1.77%/nm | 5-10 µm  (single array) | Lorentz lineshape | Yes | Yes | 0.305 nm | 100×100 µm^2^, single array |
|  | MA, OC-Hµ | 7.25%/nm | 3-10 µm  (single array) | Lorentz lineshape | Yes | Yes | 0.074 nm | 100×100 µm^2^, single array |

**Table S3** The coupling parameters of different devices were extracted based on TCMT. Unit: cm^-1^, (except *f*, *ξ* and *R^2^*)

| Device | *ω_01_* | *ω_m_* | *γ_r_* | *γ_a_* | *μ* | *γ_m_* | *Δω* | *f* | *ξ* | *R^2^* |
| --- | --- | --- | --- | --- | --- | --- | --- | --- | --- | --- |
| UC-Hµ1 | 1765.1 | 1733 | 30.9 | 85 | 23.5 | 10.2 | -32.1 | 0.4 | 2.3 | 0.99 |
| UC-Hµ2 | 1637.9 | 1730 | 26 | 67.5 | 30.5 | 12.8 | 92.1 | 0.4 | 2.4 | 0.99 |
| UC-Hµ3 | 1322.2 | 1730 | 13.8 | 29.2 | 27.6 | 11 | 407.8 | 0.5 | 2.5 | 0.97 |
| OC-1 | 1719.9 | 1730.7 | 208 | 14.2 | 8.9 | 7.8 | 10.8 | 14.6 | 1.1 | 0.95 |
| OC-2 | 1488.3 | 1732.6 | 200.2 | 12.1 | 9.7 | 9.9 | 244.3 | 16.5 | 1.0 | 0.96 |
| OC-3 | 1280.2 | 1731.8 | 159.6 | 11.2 | 8.7 | 10.1 | 451.6 | 14.3 | 0.9 | 0.97 |
| OC-Hµ1 | 1731.1 | 1729.4 | 199.6 | 26.2 | 30.9 | 4.17 | -1.7 | 7.6 | 7.4 | 0.93 |
| OC-Hµ2 | 1616.1 | 1731.8 | 278.9 | 28 | 22.1 | 6.7 | 115.7 | 10.0 | 3.3 | 0.85 |
| OC-Hµ3 | 1275.1 | 1730.2 | 153.7 | 13.7 | 35.4 | 13.1 | 455.1 | 11.2 | 2.7 | 0.81 |

**Table S4** Langmuir EXT1 fitting results for signal enhancement

| Molecule | Fingerprint peak | Parameter a | Parameter b | Parameter c | R^2^ |
| --- | --- | --- | --- | --- | --- |
| Lactic acid | 1727 cm^-1^ | 46.163 | 0.124 | 0.495 | 0.999 |
|  | 1208 cm^-1^ | 8.868 | 0.053 | 0.522 | 0.996 |
|  | 1129 cm^-1^ | 18.895 | 0.035 | 0.583 | 0.998 |
| Urea | 1659 cm^-1^ | 101.655 | 0.063 | 0.724 | 0.990 |
|  | 1621 cm^-1^ | 124.034 | 0.055 | 0.713 | 0.993 |
|  | 1449 cm^-1^ | 285.804 | 0.009 | 0.722 | 0.986 |
| BSA | 1657 cm^-1^ | 47.367 | 0.367 | -0.033 | 0.998 |
|  | 1546 cm^-1^ | 21.022 | 0.274 | -0.702 | 0.997 |
| BLG | 1641 cm^-1^ | 30.019 | 0.208 | -0.070 | 0.992 |
|  | 1545 cm^-1^ | 14.903 | 0.0478 | -0.990 | 0.999 |

**Supplementary References**

1. E. Shiles, T. Sasaki, M. Inokuti, D.Y. Smith, Self-consistency and sum-rule tests in the kramers-kronig analysis of optical-data-applications to aluminum. Phys. Rev. B **22**, 1612-1628 (1980). <https://doi.org/10.1103/PhysRevB.22.1612>
2. E.D. Palik, Handbook of optical constants of solids. (Academic press; 1998).
3. X. N. Zhang, J. Qiu, J. M. Zhao, X. C. Li, L. H. Liu, Complex refractive indices measurements of polymers in infrared bands. J. Quant. Spectrosc. Radiat. Transfer. **252**, 107063 (2020). <https://doi.org/10.1016/j.jqsrt.2020.107063>
4. H. Durmaz, Y.Y. Li, A.E. Cetin, A multiple-band perfect absorber for seira applications. Sensors and Actuators B-Chem. **275**, 174-179 (2018). <https://doi.org/10.1016/j.snb.2018.08.053>
5. H.A. Haus, Waves and fields in optoelectronics (Prentice-Hall, Inc.; 1984).
6. D. Li, H. Zhou, Z. Chen, Z. Ren, C. Xu et al., Ultrasensitive molecular fingerprint retrieval using strongly detuned overcoupled plasmonic nanoantennas. Adv. Mater. **35**, e2301787 (2023). <https://doi.org/10.1002/adma.202301787>
7. R. Adato, A. Artar, S. Erramilli, H. Altug, Engineered absorption enhancement and induced transparency in coupled molecular and plasmonic resonator systems. Nano Lett. **13**, 2584-2591 (2013). <https://doi.org/10.1021/nl400689q>
8. J. Wang, T. Weber, A. Aigner, S.A. Maier, A. Tittl, Mirror‐coupled plasmonic bound states in the continuum for tunable perfect absorption. Laser Photon. Rev. 2300294 (2023). <https://doi.org/10.1002/lpor.202300294>
9. A. Aigner, A. Tittl, J. Wang, T. Weber, Y. Kivshar et al., Plasmonic bound states in the continuum to tailor light-matter coupling. Sci. Adv. **8**, eadd4816 (2022). <https://doi.org/10.1126/sciadv.add4816>
10. Tittl, A. Leitis, M. Liu, F. Yesilkoy, D.-Y. Choi et al., Imaging-based molecular barcoding with pixelated dielectric metasurfaces. Science **360**, 1105–1109 (2018). <https://doi.org/10.1126/science.aas9768>
11. W. Yue, V. Kravets, M. Pu, C. Wang, Z. Zhao et al., Multiple-resonant pad-rod nanoantennas for surface-enhanced infrared absorption spectroscopy. Nanotechnology **30**, 465206 (2019). <https://doi.org/10.1088/1361-6528/ab3b69>
12. L. Paggi, A. Fabas, H. El Ouazzani, J.P. Hugonin, N. Fayard et al., Over-coupled resonator for broadband surface enhanced infrared absorption (seira). Nat. Commun. **14**, 4814 (2023). <https://doi.org/10.1038/s41467-023-40511-7>
13. A.A. Habeeb, H. Long, L. Bao, K. Wang, B. Wang et al., Surface plasmonic resonances and enhanced ir spectra in gzo nano-triangle arrays. Mater. Lett. **172**, 36-39 (2016). <https://doi.org/10.1016/j.matlet.2016.02.070>
14. K. Chen, R. Adato, H. Altug, Dual-band perfect absorber for multispectral plasmon-enhanced infrared spectroscopy. ACS Nano **6**, 7998–8006 (2012). <https://doi.org/10.1021/nn3026468>
15. F. Cheng, X. Yang, J. Gao, Ultrasensitive detection and characterization of molecules with infrared plasmonic metamaterials. Sci. Rep. **5**, 14327 (2015). <https://doi.org/10.1038/srep14327>
16. Lahiri, A.Z. Khokhar, R.M. De La Rue, S.G. McMeekin, N.P. Johnson, Asymmetric split ring resonators for optical sensing of organic materials. Opt. Express **17**, 1107–1115 (2009). <https://doi.org/10.1364/oe.17.001107>
17. J.M. Hoffmann, X. Yin, J. Richter, A. Hartung, T.W. Maß et al., Low -cost infrared resonant structures for surface-enhanced infrared absorption spectroscopy in the fingerprint region from 3 to 13 μm. The J. Phys. Chem. C **117**, 11311-11316 (2013). <https://doi.org/10.1021/jp402383h>
18. J.W. Petefish, A.C. Hillier, Angle-tunable enhanced infrared reflection absorption spectroscopy via grating-coupled surface plasmon resonance. Anal. Chem. **86**, 2610–2617 (2014). <https://doi.org/10.1021/ac4038398>
19. T. Wang, V.H. Nguyen, A. Buchenauer, U. Schnakenberg, T. Taubner, Surface enhanced infrared spectroscopy with gold strip gratings. Opt. Express **21**, 9005–9010 (2013). <https://doi.org/10.1364/OE.21.009005>
20. A.E. Cetin, S. Korkmaz, H. Durmaz, E. Aslan, S. Kaya et al., Quantification of multiple molecular fingerprints by dual-resonant perfect absorber. Adv. Opt. Mater. **4**, 1274-1280 (2016). <https://doi.org/10.1002/adom.201600305>
21. Y. Li, H. Yan, D.B. Farmer, X. Meng, W. Zhu et al., Graphene plasmon enhanced vibrational sensing of surface-adsorbed layers. Nano Lett. **14**, 1573–1577 (2014). <https://doi.org/10.1021/nl404824w>
22. M. Abb, Y. Wang, N. Papasimakis, C.H. de Groot, O.L. Muskens, Surface-enhanced infrared spectroscopy using metal oxide plasmonic antenna arrays. Nano Lett. **14**, 346–352 (2014). <https://doi.org/10.1021/nl404115g>
23. H. Zhou, Z. Ren, C. Xu, L. Xu, C. Lee, Mof/polymer-integrated multi-hotspot mid-infrared nanoantennas for sensitive detection of CO(2) gas. Nano-Micro Lett. **14**, 207 (2022). <https://doi.org/10.1007/s40820-022-00950-1>
24. J. Wei, Y. Li, Y. Chang, D.M.N. Hasan, B. Dong et al., Ultrasensitive transmissive infrared spectroscopy via loss engineering of metallic nanoantennas for compact devices. ACS Appl. Mater. Interfaces **11**, 47270–47278 (2019). <https://doi.org/10.1021/acsami.9b18002>
25. Z. Ren, Z. Zhang, J. Wei, B. Dong, C. Lee, Wavelength-multiplexed hook nanoantennas for machine learning enabled mid-infrared spectroscopy. Nat. Commun. **13**, 3859 (2022). <https://doi.org/10.1038/s41467-022-31520-z>
